# SRGN amplifies microglia-mediated neuroinflammation and exacerbates ischemic brain injury

**DOI:** 10.1186/s12974-024-03026-6

**Published:** 2024-01-29

**Authors:** Yi Qian, Lixuan Yang, Jian Chen, Chao Zhou, Ningning Zong, Yang Geng, Shengnan Xia, Haiyan Yang, Xinyu Bao, Yan Chen, Yun Xu

**Affiliations:** 1 Department of Neurology, Nanjing Drum Tower Hospital, Affiliated Hospital of Medical School, Nanjing University, Nanjing, 210008 China; 2 Department of Neurology, Nanjing Drum Tower Hospital, State Key Laboratory of Pharmaceutical Biotechnology and Institute of Translational Medicine for Brain Critical Diseases, Nanjing University, Nanjing, 210008 China; 3 Jiangsu Key Laboratory for Molecular Medicine, Medical School of Nanjing University, Nanjing, 210008 China; 4 Jiangsu Provincial Key Discipline of Neurology, Nanjing, 210008 China; 5 Nanjing Neurology Medical Center, Nanjing, 210008 China

**Keywords:** Ischemic stroke, Neuroinflammation, Microglia, Serglycin, CD44

## Abstract

**Background:**

Microglia is the major contributor of post-stroke neuroinflammation cascade and the crucial cellular target for the treatment of ischemic stroke. Currently, the endogenous mechanism underlying microglial activation following ischemic stroke remains elusive. Serglycin (SRGN) is a proteoglycan expressed in immune cells. Up to now, the role of SRGN on microglial activation and ischemic stroke is largely unexplored.

**Methods:**

*Srgn* knockout (KO), *Cd44*-KO and wild-type (WT) mice were subjected to middle cerebral artery occlusion (MCAO) to mimic ischemic stroke. Exogenous SRGN supplementation was achieved by stereotactic injection of recombinant mouse SRGN (rSRGN). Cerebral infarction was measured by 2,3,5-triphenyltetrazolium chloride (TTC) staining. Neurological functions were evaluated by the modified neurological severity score (mNSS) and grip strength. Microglial activation was detected by Iba1 immunostaining, morphological analysis and cytokines’ production. Neuronal death was examined by MAP2 immunostaining and FJB staining.

**Results:**

The expression of SRGN and its receptor CD44 was significantly elevated in the ischemic mouse brains, especially in microglia. In addition, lipopolysaccharide (LPS) induced SRGN upregulation in microglia in vitro. rSRGN worsened ischemic brain injury in mice and amplified post-stroke neuroinflammation, while gene knockout of *Srgn* exerted reverse impacts. rSRGN promoted microglial proinflammatory activation both in vivo and in vitro*,* whereas *Srgn*-deficiency alleviated microglia-mediated inflammatory response. Moreover, the genetic deletion of *Cd44* partially rescued rSRGN-induced excessed neuroinflammation and ischemic brain injury in mice. Mechanistically, SRGN boosted the activation of NF-κB signal, and increased glycolysis in microglia.

**Conclusion:**

SRGN acts as a novel therapeutic target in microglia-boosted proinflammatory response following ischemic stroke.

**Supplementary Information:**

The online version contains supplementary material available at 10.1186/s12974-024-03026-6.

## Background

Ischemic stroke is a worldwide disease with high mortality and disability rates [1, 2]. Current intravenous thrombolysis and mechanical thrombectomy therapies can only benefit a small fraction of patients due to the narrow time window and potential secondary damage caused by ischemia–reperfusion. Accumulating evidence has shown that microglia-mediated neuroinflammation contributes greatly to the secondary damage after stroke [3, 4]. It can lead to blood–brain barrier breakdown, neuronal damage, neurological deficits and so on [5].

After the onset of ischemic stroke, microglia are activated and release proinflammatory cytokines such as IL-1β, IL-6 and TNF-α to the ischemic brain regions [6, 7]. Meanwhile, they also recruit peripheral immune cells to brain parenchyma by releasing chemokines [8, 9]. Hyperactivated microglia and the gathered infiltrating immune cells could release more inflammatory mediators, further amplifying the inflammatory response and damaging the brain [10]. Thus, targeting microglia-mediated inflammatory cascades is the key to immunomodulatory treatment of stroke. However, firm evidence on the key factors for microglia-mediated neuroinflammation are still lacking, and the underlying mechanism is in its infancy.

To discover new molecules in controlling microglia-mediated proinflammatory response after ischemic stroke, we screened all genes encoding secretory proteins in microglia from our single-cell RNA sequencing (scRNA-seq) data in a murine model of ischemic stroke [11]. We found that *Srgn*, among the top 10 differentially expressed genes (DEGs), was increased at 3 h, 12 h and 3d after brain ischemia. Additionally, *Srgn* was expressed among different microglia subpopulations, indicating the potential vital role of *Srgn* in regulating post-stroke neuroinflammation. Serglycin (SRGN, encoded by the gene *Srgn*) is a secretory granule proteoglycan with a 17.6 kDa core protein and glycosaminoglycan (GAG) chains, and was first discovered in a rat yolk sac tumor by Oldberg et.al. in the 1980s [12]. SRGN is capable of signal transduction through the transmembrane protein receptor CD44 [13]. After activated by SRGN, CD44 can mediate a variety of signaling pathways including the NF-κB, AKT, MAPK/ERK and the β-catenin [14–17]. Previous studies showed that SRGN participated in various cancer progression including myeloid leukemia [18], multiple myeloma [19], breast cancer [20], lung cancer [21–24] and giant cell tumor of bone [25]. Also, it plays an important role in immune response. For instance, SRGN secreted by peritoneal macrophages could regulate their own TNF-α secretion under LPS stimulation [26]. In adipose tissue, immune cells-derived SRGN is linked with obesity-induced inflammation. *Srgn-*knockout (KO) mice showed a lower expression of inflammatory genes and less infiltration of proinflammatory M1 macrophages in adipose tissue [27].

Despite the vital roles of SRGN in regulating cancer progression and immune response, few studies were aimed to investigate the role of SRGN in the central nervous system [28]. Especially, the function of SRGN in ischemic stroke and microglia-mediated neuroinflammation is still unknown. In this study, we found that SRGN was remarkably elevated in the ischemic brain, and microglia were the main source of the upregulation. In addition, activated microglia secreted SRGN to activate the homeostatic state microglia, thus amplifying the post-stroke inflammation cascade. We further investigated the impact of SRGN on the outcome of ischemic brain injury and explored the underlying mechanisms regarding the actions of SRGN in ischemic stroke.

## Materials and methods

### Regents and antibodies

Recombinant mouse SRGN (rSRGN) was purchased from Cusabio (Wuhan, China). Primary antibody dilution factors and vendor information for the western blotting and IF staining protocols are listed in Additional file 1: Table S1.

### Animals

Male C57BL/6J (B6) mice were purchased from the Model Animal Center of Nanjing University (Jiangsu, China). *Srgn*-KO mice on B6 background were generated by the Cyagen Biosciences (Jiangsu, China). Male *Cd44*-KO mice on B6 background were kindly provided by Professor Sun Cheng (Nantong University). The gene knockout efficiency of *Srgn* and *Cd44* was validated prior to the formal experiments (Additional file 2: Fig. S1A, B). All mice were housed in a specific-pathogen-free (SPF) environment with a consistent 12-h light/12-h dark cycle and were given ad libitum access to food and water. All animal-based experimental protocols were sanctioned by, and executed in adherence to, the guidelines set by Animal Care Committee of Nanjing University (approval number: 2023AE01024).

### Murine model of ischemic stroke

In this study, the middle cerebral artery occlusion (MCAO) mouse model was employed as previously described [29]. Briefly, 8- to 10-week-old male mice weighing 20–25 g were anesthetized with Avertin and body temperature was maintained at 37.5 ± 0.5 °C using a controlled heating system throughout all procedures. A 6-0 silicone-coated suture (Doccol Corporation, MA, USA) was carefully inserted into the right internal carotid artery until reaching the origin of the MCA, ensuring proper occlusion. Cerebral blood flow was monitored using Laser Doppler flowmetry (Perimed Corporation, Stockholm, Sweden). After 60 min of occlusion, the filament was removed to initiate cerebral blood reperfusion. Once reperfusion was confirmed, all incisions were meticulously closed, and the subjects were monitored in a recovery cage until they regained alertness. Sham procedures followed similar steps, with the omission of MCA suture insertion. The animals were randomly assigned to the MCAO or the sham groups by a lottery box.

### Quantification of brain infarct volume

Infarct volume was assessed either 24 h or 72 h post MCAO surgery using 2,3,5-triphenyltetrazolium chloride (TTC) stain (Sigma-Aldrich, Darmstadt, Germany) as previously described [30]. Briefly, mice were euthanized, and the brains were excised and positioned in mouse brain matrix slicers. The brains were then briefly frozen at − 20 °C for 15 min before being sectioned into 1-mm-thick coronal slices. These slices were subsequently stained with 2% TTC for 15 min. Regions devoid of red staining indicated infarcted tissues. Digital photographs of the slices were captured, and infarct areas were quantitatively analyzed using Image J software. The percentage of infarct volume was determined with the formula: Infarct volume = (contralateral area − ipsilateral non-infarct area)/2 × contralateral area × 100%.

### Neurological behavior tests

The neurological functions were evaluated by the modified neurological severity score (mNSS) and grip strength tests as previously described with minor adjustments [29]. The mNSS test was conducted on mice 24 h or 72 h after MCAO. The mNSS test evaluates various domains: sensory, motor, reflex, and balance. Scoring for this test ranges from 0 to 12, where higher scores indicate more pronounced neurological impairments. The grip strength test was employed to assess the forelimb muscle strength of the mice. Each mouse’s tail was lifted to force it to grasp the platform of the grip strength meter (Ugo Basile, Gemonio, Italy). Subsequently, the mouse was pulled away from the platform in a linear manner. The maximal grip strength exerted by the forelimbs during this process was documented.

### Drug preparation and administration

The rSRGN was dissolved in 50% glycerol to a final concentration of 2.5 mg/mL. Mice of the same genotype were randomly assigned to two groups, either administered with rSRGN or the solvent control (50% glycerol) within 10 min after MCAO.

### Stereotaxic intracranial injection

Stereotaxic intracranial injection was performed as previously described [9]. In brief, mice were anesthetized and securely positioned in a stereotaxic frame. An incision was made to expose the skull, followed by drilling at the designated coordinates. The targeted site for the lateral ventricle injection was determined based on a standardized brain atlas with the following coordinates: 1.5 mm lateral to the midline, 1.1 mm posterior to bregma, and 2.0 mm ventral from bregma). A volume of 2 μl rSGRN (2.5 mg/mL) or 50% glycerol was precisely injected into the lateral ventricle. To prevent reflux of the infused solution, the microelectrode was kept in place for an additional 10 min post-infusion. The incision was subsequently sutured, and the mice were allowed to recover on a thermostatic heat.

### Microglial depletion

For the purpose of depleting microglia, mice were administered either a control diet (AIN-76A, SYSE Bio, China) or a diet containing PLX5622 (based on AIN-76A with 1200 ppm PLX5622, SYSE Bio) [31]. Dietary treatment was commenced 14 days prior to surgical procedures and was maintained until the conclusion of the experiments. Typically, one adult mouse consumes approximately 3.5 g of this chow diet daily.

### Cell culture

Primary microglia were harvested and cultured as described previously [32]. Briefly, neonatal C57BL/6J mouse cortices (P2) were dissociated and cultured in DMEM with 10% FBS and 1% antibiotics (100 U/ml penicillin and 100 μg/ml streptomycin). Culture medium was replaced every other day. After 10–11 days of culture, primary microglia were isolated through a gentle shaking method (250 rpm, 10 min) and seeded onto fresh plates. The resultant primary microglial cultures exhibited a purity exceeding 95%. BV2 cells were purchased from the China Infrastructure of Cell Line Resources (Beijing, China) and were cultured using the same medium employed for primary microglia.

Primary neuronal cells were harvested from mouse embryos (E15) as described previously [32]. Briefly, mouse embryonic cortices were dissected under the guidance of an anatomical microscope using Hanks' Balanced Salt Solution (HBSS). After digestion, the tissue suspension was passed through a 40-μm cell strainer to achieve a single-cell suspension. Cells were then seeded onto poly(l-lysine)-coated culture plates at a density of 5 × 10^5 cells/mL in neurobasal medium enriched with 2% B27 supplement. Every alternate day, half of the culture medium was refreshed. On the seventh day in vitro (DIV7), neurons were retrieved. The resulting neuronal cultures achieved a purity exceeding 95% for subsequent experiments.

Neuron–microglia co-culture: Twenty-four hours post-rSRGN treatment, microglia were collected, resuspended in neurobasal media (Gibco) and replated among neurons in confocal dishes. Another 24 h later, the co-cultures were washed three times with phosphate-buffered saline (PBS) and fixed by 4% paraformaldehyde (PFA) for further study.

### Oxygen–glucose deprivation/reoxygenation (OGD/R)

OGD/R was performed as previously described [33]. In brief, microglia were transitioned to deoxygenated glucose-free DMEM (Invitrogen, CA, USA) and then incubated in an anaerobic chamber filled with a gas mixture of 5% CO_2_ and 95% N2 for a period of 3 h at 37 °C. Following the OGD exposure, the cells were returned to normal culture conditions with DMEM supplemented with 10% FBS and high glucose, and incubated at 37 °C in a humidified atmosphere of 5% CO_2_ for an additional 6 h.

### Glucose consumption

Microglial glucose metabolism was evaluated as previously described [34]. Before the assay, BV2 cells were incubated in media with rSRGN for 24 h. Then, glucose uptake was measured using glucose assay kit (Solarbio® BC2500) according to the manufacturer’s instructions.

### Quantification of lactate levels

Initially, BV2 cells were treated with rSRGN for 24 h. Following stimulation, the cell culture supernatant was collected. The quantity of lactate present in the supernatant was determined using a L-lactic acid (L-LA) Content Assay Kit (Solarbio, Beijing, China) according to the manufacturer’s instructions.

### Flow cytometry

Mice underwent transcardial perfusion with 50 mL of cold PBS supplemented with 5 IU/mL heparin. Subsequently, brains were swiftly excised and immersed in 1 × HBSS fortified with 25% glucose and HEPES buffer. Tissue was minced and subjected to gentle mechanical dissociation in DMEM media. The resulting tissue–media mixture was filtered through a 70-µm mesh to achieve a single-cell suspension. This suspension was subsequently layered onto a 30–70% percoll gradient (GE Healthcare BioSciences, NJ, USA) and centrifuged at 2500 rpm for 20 min. Cells localized at the gradient interface were harvested and stained with fluorophore-conjugated antibodies targeting mouse CD45, CD11b, Ly6G, CD44 and IL-1β. Flow cytometric analysis was conducted using a FACS system from BD Biosciences (Carlsbad, CA, USA).

### Co-immunoprecipitation (Co-IP)

For immunoprecipitation, a total of 500 μg proteins were pretreated with 1 μg anti-SRGN antibody or normal mouse IgG overnight at 4 °C. Then, the proteins were incubated with pre-cleaned protein A/G agarose beads (Millipore) at 4 °C for 2 h. After three times of washing, co-immunoprecipitated proteins were eluted with 1 × western blot loading buffer and processed by western blotting using corresponding antibodies.

### Western blotting

Brain tissue and cells were lysed using RIPA lysis buffer (Beyotime, Shanghai, China) containing 1% protease inhibitor cocktail (Sigma-Aldrich). Protein concentrations in the lysates were quantified using the bicinchoninic acid assay (Beyotime). Samples were then subjected to SDS-PAGE on 8–12% polyacrylamide gels, and proteins were transferred to PVDF membranes. Following a 2-h block in 5% non-fat dry milk at room temperature, the membranes were probed with specific primary antibodies overnight at 4 °C. Subsequently, the membranes were incubated with the appropriate horseradish peroxidase (HRP)-conjugated secondary antibodies. After three washes in PBS-T, protein bands were visualized using the ECL Detection Kit (Bioworld). Images of the bands were captured with the Gel-Pro system (Tanon Technologies, Shanghai, China) and then quantitatively analyzed using Image J software.

### Enzyme linked immunosorbent assay (ELISA)

The concentration of SRGN in both mouse serum and cerebral cortex homogenates was determined using an ELISA kit (SAB, China), strictly adhering to the manufacturer's protocol. Optical density (OD) values at a wavelength of 450 nm were recorded using a Tecan microplate reader (Tecan, Switzerland), facilitating the quantification of SRGN levels.

### Immunofluorescence staining

Mice were anesthetized and subsequently perfused transcardially with PBS followed by 4% PFA. The mice brains were carefully extracted, post-fixed, and dehydrated. Subsequently, brain frozen sections of 20 μm thickness were prepared. Brain sections were permeabilized with 0.25% Triton X-100 for 20 min and blocked with 2% bovine serum albumin (BSA) for 2 h at room temperature. The sections were then incubated with primary antibodies at 4 °C overnight. Following thorough washing with PBS, the slices were incubated with appropriate secondary antibodies for 1 h at room temperature. Nuclei were counterstained using DAPI (Beyotime) for 15 min. Fluorescent imaging was taken by an Olympus FV3000 confocal microscope (Olympus, Japan).

### Transcriptome sequencing

Total RNA was extracted from primary microglia, both stimulated and unstimulated by rSRGN, using TRIzol (Invitrogen). The quality and quantity of RNA were assessed using the NanoDrop 2000 spectrophotometer (Thermo), while RNA integrity was determined using the Agilent 2100 Bioanalyzer (Agilent Technologies, CA, USA). RNA-sequencing transcriptome analysis and subsequent downstream analysis were performed by OE Biotech Co., Ltd. (Shanghai, China). DEGs were selected based on the criteria of |LogFC|≥ 2 and a *p* value < 0.05. Subsequently, Gene Ontology (GO) analysis and Kyoto Encyclopedia of Genes and Genomes (KEGG) analysis were applied to elucidate the functional roles of these differentially expressed mRNAs, followed by further analysis.

### Quantitative PCR (qPCR)

Total RNA was extracted from primary microglia and brain tissue with AG RNAex Pro Reagent (AG, China) according to the manufacturer's instruction. The extracted RNA was reverse transcribed to cDNA utilizing the PrimeScript RT Reagent Kit (Vazyme, Nanjing, China). Real-time qPCR was performed using SYBR Green qPCR Master Mix (AG) with a Step One Plus PCR system (Applied Biosystems, CA, USA). The sequences of primers used are listed in Additional file 3: Table S2.

### Statistical analysis

The normality of data was evaluated with the Shapiro–Wilk test. All results are presented as standard error of mean (mean ± SEM). Statistical differences were evaluated using Student's *t*-tests for two groups, one-way ANOVA followed by the Bonferroni’s post hoc test for three or more groups. The statistical analysis was performed by GraphPad Prism software (Version 8.0). *P* < 0.05 was considered as statistically significant.

## Results

### SRGN is remarkably elevated after focal ischemia, especially in microglia

**Fig. 1:**
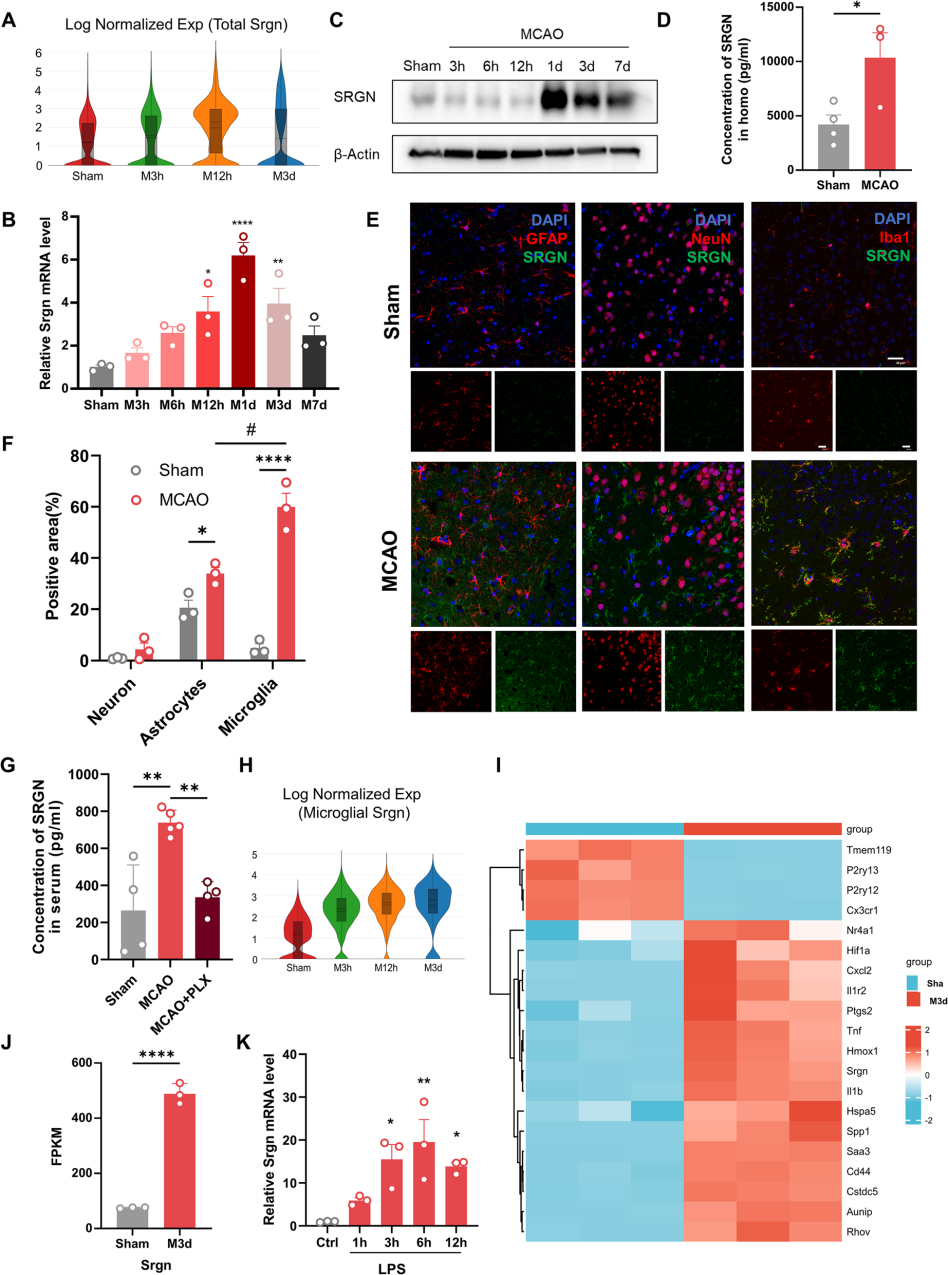
SRGN is remarkably elevated after focal ischemia, especially in microglia. **A** Violin plot showing the total expression of *Srgn* gene from the ischemic brain at different time points (Sham, 3 h, 12 h, 3d) after MCAO according to the scRNA-seq data from our previous work. **B** The qPCR analysis of *Srgn* mRNA levels at different time points (Sham, 3 h, 6 h, 12 h, 1d, 3d, 7d) after MCAO. **C** Representative immunoblot image showing the protein level of SRGN at different time points (Sham, 3 h, 6 h, 12 h, 1d, 3d, 7d) after MCAO. β-Actin served as the loading control. **D** The statistical analysis of brain homogenate SRGN level after MCAO 1 day. *n* = 3 ~ 4 mice per group. **E** Representative immunofluorescence images of SRGN expression in cortex neurons (NeuN^+^), astrocytes (GFAP^+^) and microglia (Iba1^+^) of mice after MCAO 1 day. Scale bar, 30 μm. **F** Quantification of co-localization of SRGN with NeuN, GFAP or Iba1 in cortex of mice 1 day after MCAO. *n* = 3 mice per group. **G** The statistical analysis of serum SRGN level at MCAO 1 day with or without the pretreatment of microglia scavenger PLX5622. **H** Violin plot showing the microglial expression of *Srgn* gene from the ischemic brain tissue at different time points (Sham, 3 h, 12 h, 3d) after MCAO according to the scRNA-seq data from our previous work. **I** Heat map showing part of the DEGs of sorted microglia from the ischemic brain tissue after MCAO 3 days according to the bulk-RNA seq data from our previous work. **J** The FPKM of *Srgn* according to the bulk-RNA seq data from our previous work. **K** The qPCR analysis of *Srgn* mRNA levels in primary microglia at different time points (0 h, 1 h, 3 h, 6h, 12 h) after LPS (100 ng/ml) stimulation. Data represented as mean ± SEM, * *p* < 0.05, ** *p* < 0.01, ****p < 0.0001, #*p* < 0.05

To explore the potential key molecules underlying ischemic stroke, single-cell RNA-sequence was conducted at before, 3 h, 12 h, and 3d after MCAO. We identified that *Srgn* was upregulated, especially at 12 h post-stroke, among the top 10 DEGs (Fig. 1A). The expression of SRGN in the ischemic brain was also verified by qPCR and immunoblot assays. Our results demonstrated that SRGN was upregulated in the ischemic mouse brains, peaking at 1 day after MCAO (Fig. 1B, C). By ELISA assay, we also detected an increasing secretion of SRGN in the homogenate of ischemic brain tissue at 1d after MCAO (Fig. 1D). Then, we determined the contribution of different cell types to the upregulation of SRGN. Unsupervised clustering and cell type annotation of our scRNA-seq demonstrated that *Srgn* gene was mainly expressed in microglia and various types of peripherally infiltrated immune cells, like macrophage and T cells (Additional file 4: Fig. S2A). Given that microglia are the predominant resident immune cells in the brain and that SRGN expression peaked at 12–24 h after MCAO, by which time only a few peripherally immune cells would have infiltrated, we assumed that microglia were the main contributors to the increase of SRGN following stroke during the early stage. Immunostaining confirmed that SRGN was mainly colocalized with microglia (Iba1^+^), mildly in astrocytes (GFAP^+^) and endothelial cells (Cd31^+^), but not in neurons (NeuN^+^), and the change of SRGN expression before and after MCAO was most dramatic in microglia (Fig. 1E, F, Additional file 5: Fig. S3A, B). Consistently, when microglia were deleted by PLX5622, a CSF1R inhibitor [31], serum SRGN level after MCAO was significantly decreased (Fig. 1G). According to the scRNA-seq data, microglial *Srgn* was upregulated since 3 h after MCAO (Fig. 1H). In another bulk-RNA seq of our team [35], microglia were sorted from ischemic brain tissue at different time points after MCAO. Consistent with the scRNA-seq results, the differential gene expression analysis of the bulk-RNA seq also identified *Srgn* gene, which was significantly increased within 3 days after MCAO (Fig. 1I, J). Collectively, we confirmed the upregulation of SRGN after ischemic stroke, which was predominantly originated from microglia.

In vitro, we found that lipopolysaccharide (LPS) stimulation could induce the expression of microglial *Srgn* remarkably (Fig. 1K). Interestingly, we did not observe the upregulation of *Srgn* in microglia exposed to OGD/R model (Additional file 4: Fig. S2B), an in vitro model of ischemia, indicating that *Srgn* upregulation in microglia might be due to the activation of toll-like receptor (TLR) by damage-associated molecular patterns (DAMPs).

### SRGN aggravates ischemic brain injury

**Fig. 2:**
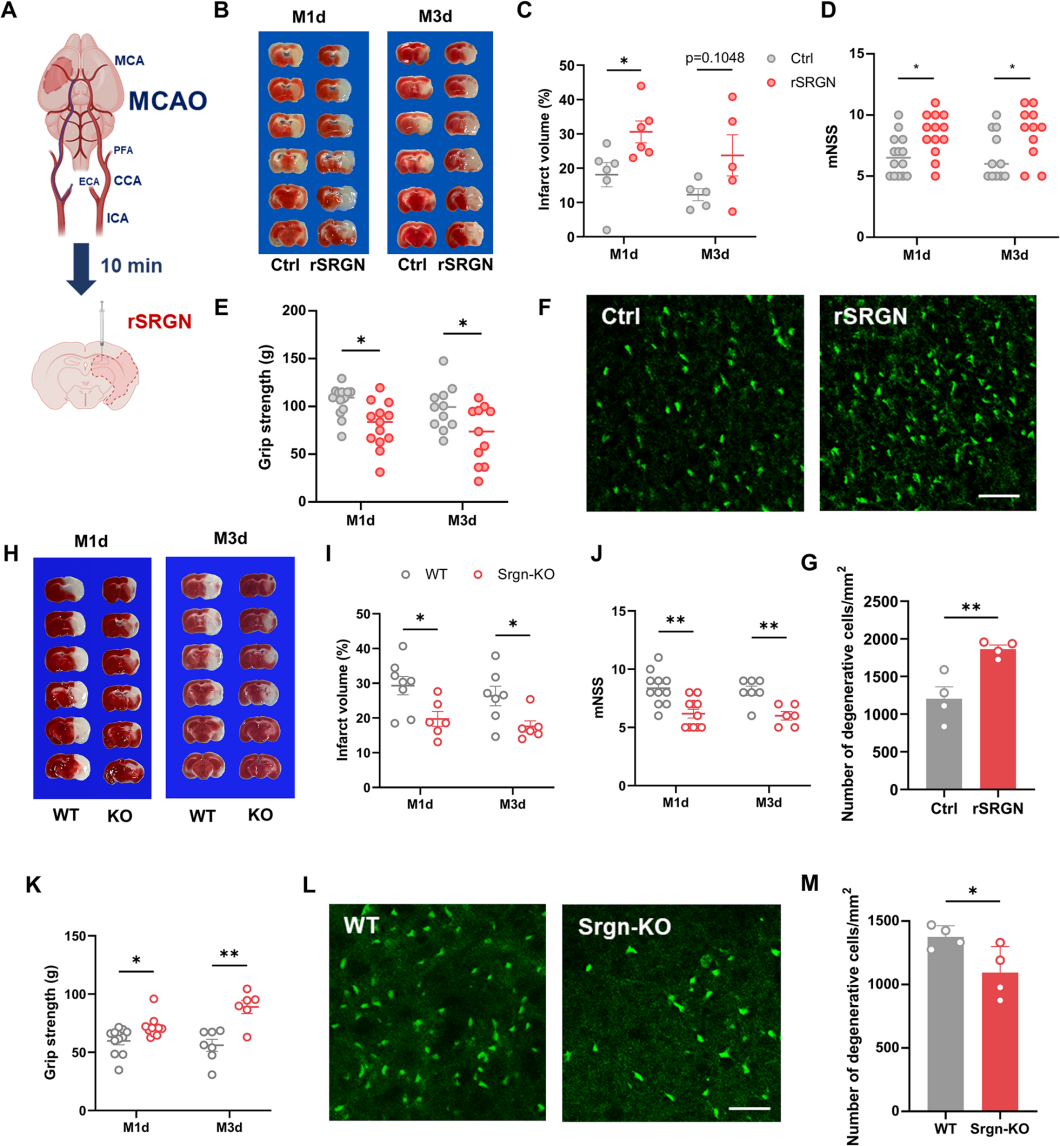
SRGN aggravates ischemic brain injury. **A** Flow chart demonstrating the operation of MCAO and the following rSRGN injection. **B**, **C** Representative images of TTC-stained brain sections (**B**) with the quantification of infarct volume (**C**) at 1 day or 3 days after MCAO and injection of control solvent (50% glycerol) or rSRGN (2.5 mg/ mL). *n* = 5 ~ 6 mice per group. **D**, **E** The modified neurological severity score (mNSS) (**D**) and grip strength (**E**) at 1 day or 3 days after MCAO. *n* = 13 ~ 14 mice for groups at 1 day and *n* = 11 mice for groups at 3 days after MCAO. **F**, **G** Representative immunofluorescence images (**F**) and quantification (**G**) of neuronal death based on FJB staining assay in the ipsilateral hemisphere of ischemia brains of control solvent or rSRGN-injected mice at 1 day after MCAO. Scale bar, 50 μm. **H**, **I** Representative images of TTC-stained brain sections (**H**) from Srgn-KO mice and their wild-type (WT) littermates with the quantification of infarct volume (**I**) at 1 day after MCAO. *n* = 8 mice for the WT group and *n* = 6 mice for the Srgn-KO group. **J**, **K** The modified neurological severity score (mNSS) (**J**) and grip strength (**K**) at 1 day or 3 days after MCAO. *n* = 11 mice for groups at 1 day and *n* = 6 ~ 7 mice for groups at 3 days after MCAO. **L**, **M** Representative immunofluorescence images (**L**) and quantification (**M**) of neuronal death based on FJB staining assay in the ipsilateral hemisphere of ischemia brains of WT controls or Srgn-KO mice at 1 day after MCAO. Scale bar, 50 μm. Data represented as mean ± SEM, * *p* < 0.05, ** *p* < 0.01

In order to clarify the impact of SRGN on the outcome of ischemic stroke, rSRGN or the solvent control were injected into the lateral ventricles of mice immediately after the MCAO surgery (Fig. 2A). Cerebral infarct volume was measured by TTC staining at 1d and 3d after MCAO. As a result, exogenous injection of rSRGN led to an enlarged infarct volume than solvent controls (Fig. 2B, C). Sensorimotor deficits were evaluated by mNSS score and grip strength test at 1d and 3d after MCAO. We demonstrated that rSRGN-injected mice exhibited more severe sensorimotor deficits, as indicated by a higher mNSS score and lower grip strength than solvent controls (Fig. 2D, E). Besides, the FJB staining of brain slices illustrated that rSRGN induced a significant loss of normal neurons (Fig. 2F, G). To further confirm the importance of SRGN in ischemic stroke, *Srgn*-KO mice were generated. The *Srgn-*KO mice and their wild-type counterparts were subjected to MCAO operation. The regional cerebral blood flow (CBF) was unaltered during the stroke course after *Srgn* gene knockout (Additional file 6: Fig. S4A, B). Our results showed that *Srgn*-knockout could improve the outcome of ischemic stroke, as indicated by the decreased infarct volume (Fig. 2H, I), lower mNSS score and higher grip strength (Fig. 2J, K). Also, the loss of normal neurons was alleviated (Fig. 2L, M). Taken together, our results demonstrated that SRGN aggravated ischemic brain injury.

### SRGN amplifies microglia-mediated inflammation via CD44 after ischemic stroke

#### CD44 receptor is upregulated in microglia after ischemic stroke

**Fig. 3:**
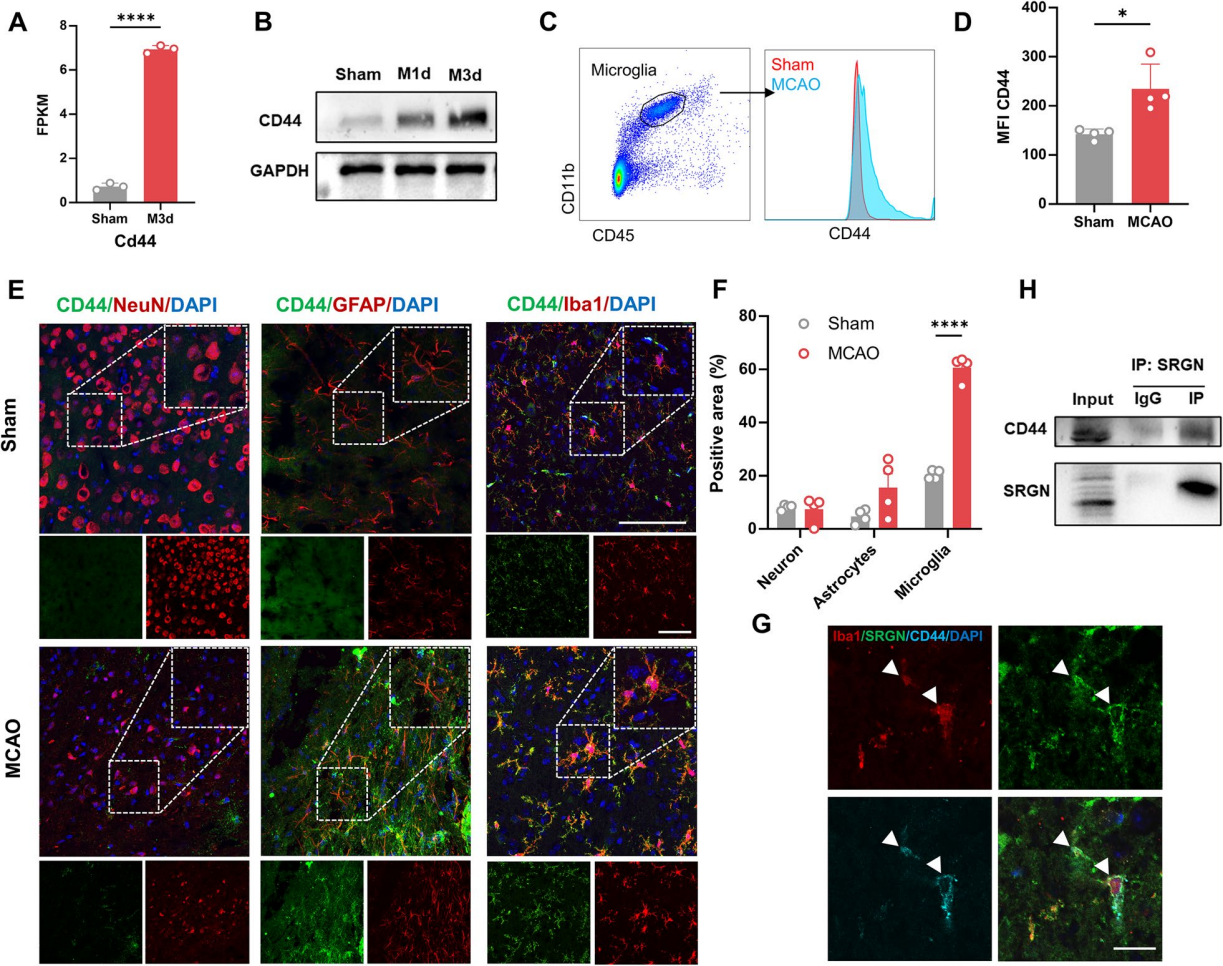
CD44 receptor is upregulated in microglia after ischemic stroke. **A** The FPKM of *Cd44* according to the bulk-RNA seq data from our previous work. **B** Representative immunoblot image showing the protein level of CD44 from ischemic brain tissue at different time points (Sham, 1d, 3d) after MCAO. GAPDH served as the loading control. **C**, **D** Flow cytometry strategy labeling microglial CD44 (C) and the quantification of mean fluorescence intensity (MFI) of CD44 in microglia from ischemic brain tissue 1 day after MCAO (D). *n* = 4 mice per group. **E** Representative immunofluorescence images of CD44 expression in cortex neurons (NeuN^+^), astrocytes (GFAP^+^) and microglia (Iba1^+^) from mice after MCAO 1 day. *n* = 4 mice per group. Scale bar, 100 μm. **F** Quantification of co-localization of CD44 with NeuN, GFAP or Iba1 in cortex of mice 1 day after MCAO. **G** Representative immunofluorescence image of co-localization of Iba1 (red), SRGN (green) and CD44 (blue). Scale bar, 40 μm. **H** Co-immunoprecipitation analysis of SRGN and CD44. Data represented as mean ± SEM, * *p* < 0.05, ****p < 0.0001

SRGN is identified as a secreted protein, which exerts its function mainly through binding with its receptor, and CD44 (encoded by the gene *Cd44*) is a well-recognized receptor of SRGN [17, 23, 36]. A few studies mentioned that CD44 was induced under brain ischemia [37, 38]. In line with these reports, our previous bulk-RNA seq [35] also saw a notable increase in *Cd44* gene expression (Fig. 3A). The immunoblot assay confirmed that CD44 was significantly upregulated 1d and 3d after MCAO (Fig. 3B). Further, using flow cytometry (Fig. 3C, D) and immunostaining (Fig. 3E, F), we found that CD44 was mainly localized in Iba1^+^ microglia. In addition, co-staining of SRGN, CD44 and Iba1 demonstrated that CD44 was colocalized with SRGN on microglia (Fig. G). Moreover, Co-IP confirmed the binding of microglial SRGN and CD44 in vitro (Fig. 3H). These results suggested that CD44 was remarkably upregulated in microglia after stroke and might mediate the function of SRGN.

#### SRGN induces microglial proinflammatory activation and amplifies LPS-induced inflammation in vitro

**Fig. 4:**
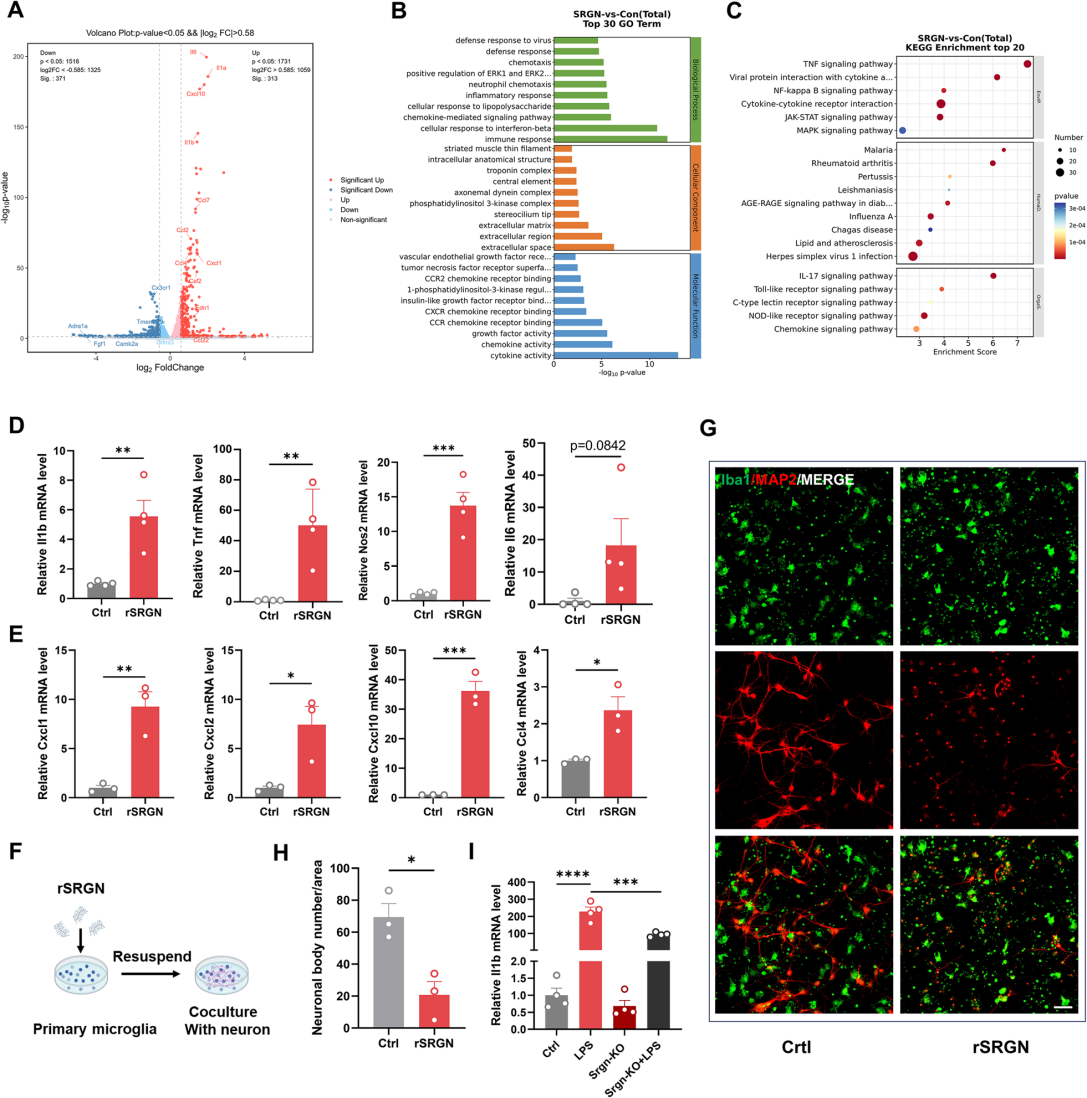
SRGN induces microglial proinflammatory activation and amplifies LPS-induced inflammation in vitro. **A** Volcano plot showing the DEGs between the control and rSRGN-treated primary microglia. **B** GO analysis of the DEGs between the control and rSRGN-treated primary microglia. Top 30 GO terms were listed. **C** KEGG enrichment of the DEGs between the control and rSRGN-treated primary microglia. Top 20 KEGG terms were listed. **D** The qPCR analysis of *Il1b*, *Tnf*, *Nos2* and *Il6* mRNA levels in primary microglia treated with rSRGN (50 ng/ mL) for 6 h. **E** The qPCR analysis of *Cxcl1*, *Cxcl2*, *Cxcl10* and *Ccl4* mRNA levels in primary microglia treated with rSRGN (50 ng/ mL) for 6 h. **F** Flow chart demonstrating the co-culture procedure (Created with BioRender.com). Primary microglia were treated with rSRGN (50 ng/ mL) or control solvent for 6 h. Then, these microglia were collected and resuspended with neuron culture medium. Afterwards, microglia were replated among neurons and the co-culture system were allowed to grow for another 24 h. **G** Representative immunofluorescence images showing the microglia (Iba1^+^, green)—neurons (MAP2^+^, red) co-culture. Scale bar, 50 μm. **H** The quantification of living neuronal bodies from the co-culture system. **I** The qPCR analysis of *Il1b* mRNA level in primary microglia extracted from neonatal Srgn-KO mice or their WT littermates, with or without LPS (100 ng/ml, 6 h) stimulation. Data represented as mean ± SEM, * *p* < 0.05, ** *p* < 0.01, *** *p* < 0.001, **** *p* < 0.0001

In order to reveal the influence of SRGN on the activation status of microglia, RNA transcriptome sequencing was performed to detect the different patterns of gene expression between the control primary microglia and the rSRGN-treated microglia (Fig. 4A). GO analysis (Fig. 4B) and KEGG enrichment (Fig. 4C) of the top DEGs indicated that rSRGN exerted an apparent impact on immune response, inflammatory response and chemotaxis of microglia. SRGN influenced several immune response-associated signaling pathways, including TNF signaling pathway, NF-kappa B signaling pathway, JAK–STAT pathway and MAPK pathway. Subsequently, the impact of SRGN on microglia-associated inflammation was examined. Our results illustrated that microglia treated with rSRGN displayed a more pronounced proinflammatory phenotype, as evidenced by the elevated mRNA level of *Il1b*, *Tnf*, *Nos2* and *Il6*, along with the upregulated mRNA level of chemotaxis factors including *Cxcl1*, *Cxcl2*, *Cxcl10* and *Ccl4* (Fig. 4D, E). Apart from the classical division of microglial phenotype [proinflammatory (M1) and anti-proinflammatory (M2) [39], our scRNA-seq analysis of microglia subclusters revealed that microglia can be divided into ischemic core-associated microglia and ischemic penumbra-associated microglia in ischemic stroke [11]. The former subcluster was thought to be destructive and was marked by *Lgals3*, *Plau*, *Srxn1*, *Ankrd33b*, *Edn1* et al., while the latter subcluster was less proinflammatory and was marked by *Pik3ip1*, Cd300lf, *Gpr65*, *Ms4a6c*, *Cep152* et al. Intriguingly, treatment with rSRGN led to a remarkable increase in *Srxn1* and *Edn1* (Additional file 7: Fig. S5A), while there was an obvious decrease in *Gpr65* and *Ms4a6c* (Additional file 7: Fig. S5B). It meant that rSRGN promoted microglia to transit towards ischemic core-related phenotype. Additionally, we co-cultured rSRGN-stimulated microglia and cortical neurons to see whether SRGN aggravated the neurotoxicity of microglia (Fig. 4F). As expected, microglia treated with rSRGN caused more neuronal death in the co-culture system compared with the control group (Fig. 4G, H).

Moreover, primary microglia from *Srgn-*KO mice and WT mice were cultured to observe the impact of *Srgn* gene knockout on microglial inflammatory response. It turned out that knockout of microglial *Srgn* blocked the elevation of *Il1b* induced by LPS (Fig. 4I). Overall, these results confirmed that SRGN promoted the immune response of microglia and it might act as an intermedia to amplify the microglial inflammatory response induced by LPS.

#### SRGN amplifies post-stroke neuroinflammation in vivo

**Fig. 5:**
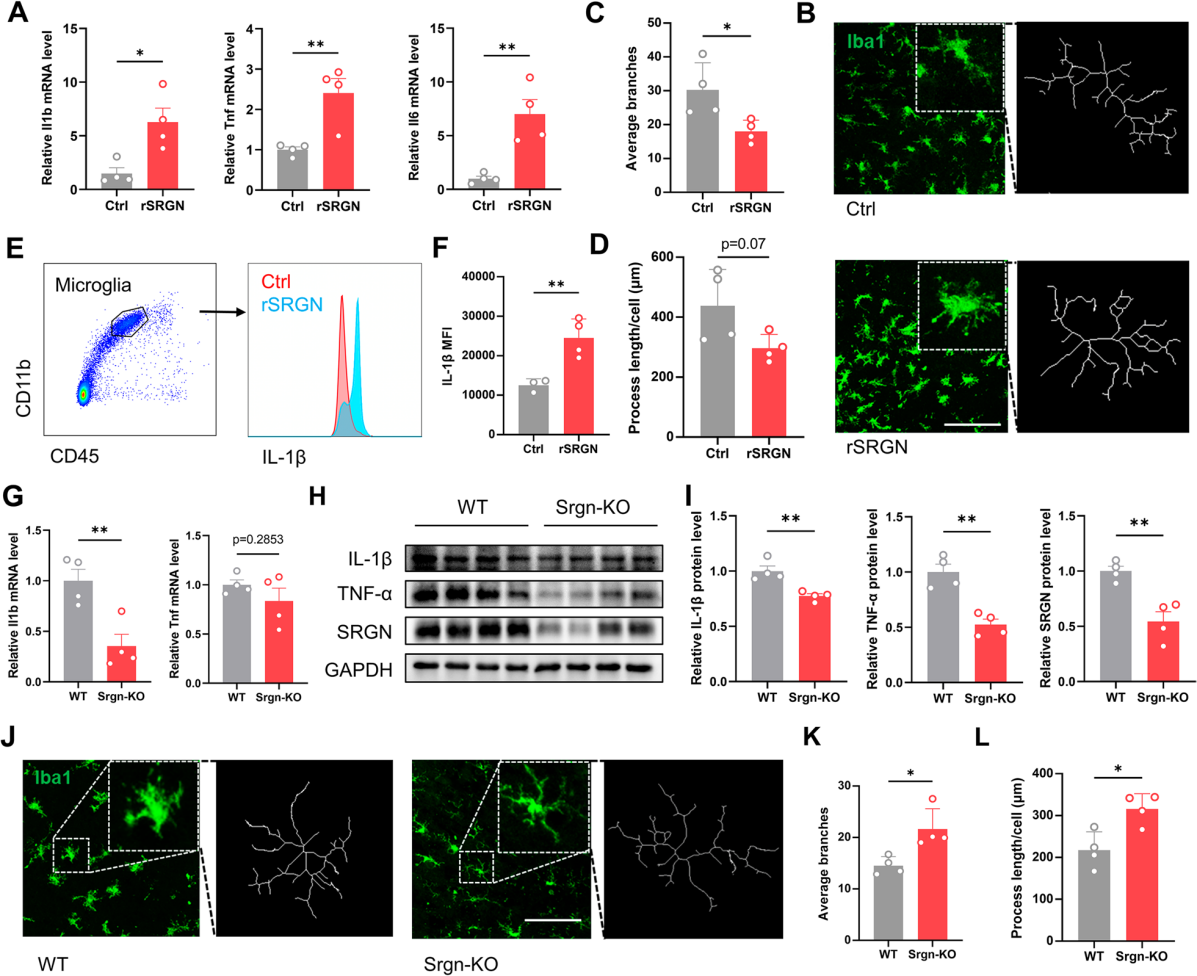
SRGN amplifies post-stroke neuroinflammation in vivo*.* **A** The qPCR analysis of *Il1b*, *Tnf* and *Il6* mRNA levels of mice brain tissues 1 day after MCAO, injected with rSRGN or control solvent. **B** Representative immunofluorescence images showing the morphology of microglia (Iba1^+^, green) in the ipsilateral hemisphere of ischemia mice brains 1 day after MCAO, injected with rSRGN (2.5 mg/ mL) or control solvent. *n* = 4 mice per group. Scale bar, 100 μm. **C**, **D** Quantification of the average branches (**C**) and process length (**D**) of microglia based on the images in **B**. **E**, **F** Flow cytometry strategy labeling microglial IL-1β (**E**) and the quantification of IL-1β MFI (**F**) in the ischemic mice brains 1 day after MCAO, injected with rSRGN (2.5 mg/ mL) or control solvent. *n* = 3 ~ 4 per group. **G** The qPCR analysis of *Il1b* and *Tnf* mRNA levels of brain tissue from Srgn-KO mice and their WT littermates 1 day after MCAO. **H**, **I** Representative immunoblot images (**H**) and quantification (**I**) of the protein level of IL-1β, TNF-α and SRGN from brain tissue of Srgn-KO mice and their WT littermates 1 day after MCAO. GAPDH served as the loading control. *n* = 4 mice per group. **J** Representative immunofluorescence images showing the morphology of microglia (Iba1^+^, green) from Srgn-KO mice and WT counterparts 1 day after MCAO. *n* = 4 mice per group. Scale bar, 100 μm. **K**, **L** Quantification of the average branches (**K**) and process length (**K**) of microglia based on the images in **J**. Data represented as mean ± SEM, * *p* < 0.05, ** *p* < 0.01

The impact of SRGN on post-stroke neuroinflammation in vivo, especially on the microglia-associated inflammation, was further explored. Firstly, we examined the gene expression of proinflammatory cytokines in the ischemic penumbra. After MCAO, mice injected with rSRGN showed a higher expression of *Il1b*, *Tnf* and *Il6* and compared with the solvent controls (50% glycerol) (Fig. 5A). Afterwards, we evaluated the microglia morphology in MCAO brain slices. Compared with the control group, microglia from the rSRGN-injected mice displayed a more ameboid shape, with rounder cell bodies, more scarce dendrites and shorter branch length (Fig. 5B–D). Moreover, flow cytometry analysis confirmed that rSRGN increased the production of proinflammatory IL-1β in CD45^int^CD11b^+^ microglia (Fig. 5E, F). Consistent with the upregulation of chemokines induced by rSRGN in microglia, flow cytometry also indicated that SRGN led to increased infiltration of peripheral macrophages (CD45^high^ CD11b^+^) and neutrophils (CD45^high^ CD11b^+^ Ly6G^+^) (Additional file 8: Fig. S6A-D). When SRGN was depleted, the expression level of proinflammatory cytokines after MCAO was decreased (Fig. 5G–I). Also, microglia shifted towards a more ramified shape (Fig. 5J–L). Taken together, SRGN activated microglia and amplified neuroinflammation after stroke.

### SRGN exacerbates microglial inflammation in a CD44-dependent manner

**Fig. 6:**
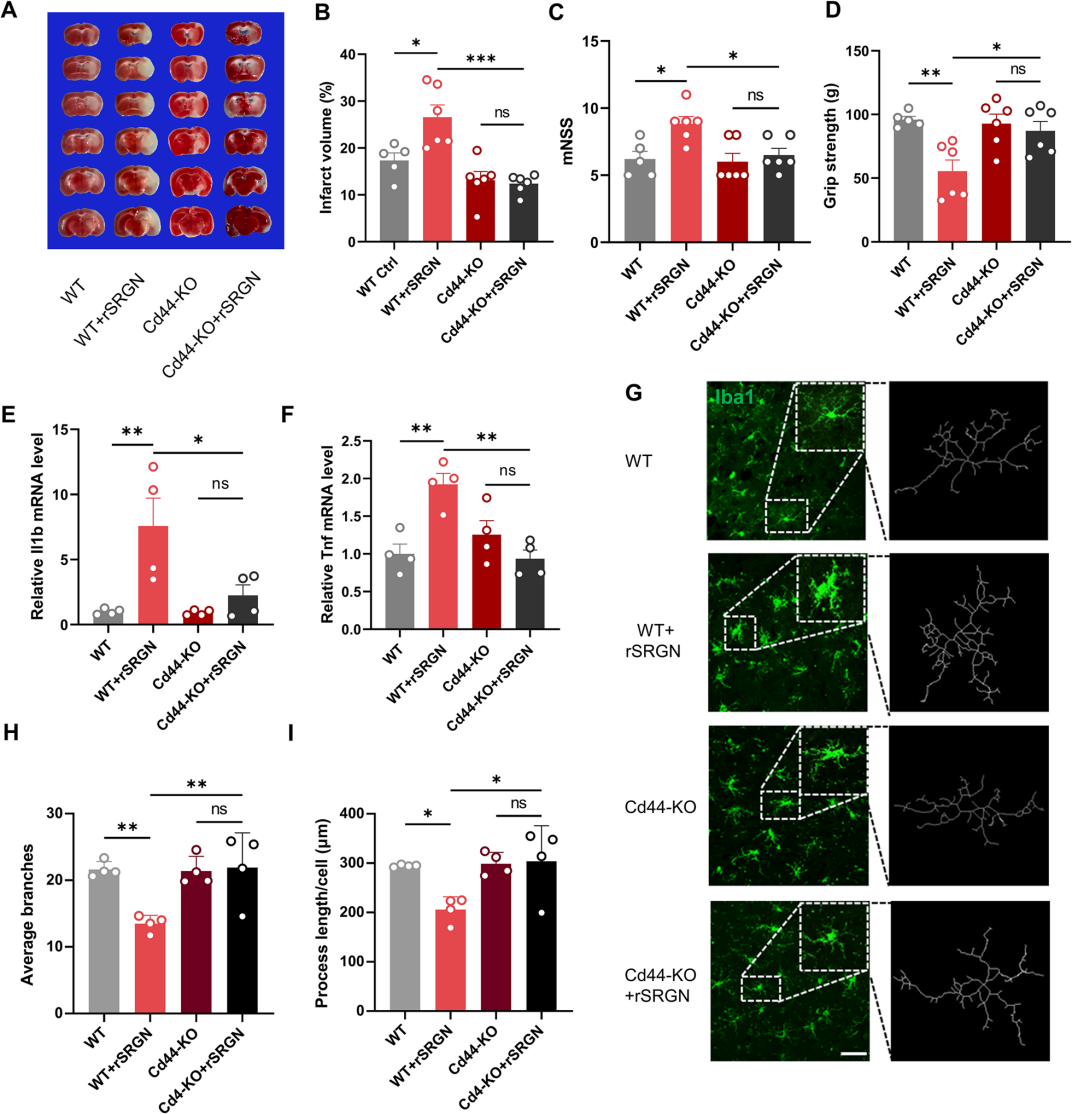
SRGN exacerbates microglial inflammation in a CD44-dependent manner. **A**, **B** Representative images of TTC-stained brain sections (**A**) with the quantification of infarct volume (**B**) at 1 day after MCAO. *Cd44*-KO mice and their WT littermates were injected with rSRGN (2.5 mg/ mL) or control solvent. *n* = 5 ~ 6 mice per group. **C**, **D** The mNSS (C) and grip strength (D) performance of *Cd44*-KO mice and their WT littermates at 1 day after MCAO. *n* = 5 ~ 6 mice per group. **E**, **F** The qPCR analysis of *Il1b* and *Tnf* mRNA levels from brain tissue of *Cd44*-KO mice and their WT littermates at 1 day after MCAO, injected with rSRGN (2.5 mg/ mL) or control solvent. **G** Representative immunofluorescence images showing the morphology of microglia (Iba1^+^, green) in ischemic brains of WT mice injected with glycerol/ rSRGN and *Cd44*-KO mice injected with rSRGN 1 day after MCAO. **H**, **I** Quantification of the average branches (**H**) and process length (**I**) of microglia based on the images in **G**. Data represented as mean ± SEM, * *p* < 0.05, ** *p* < 0.01, *** *p* < 0.001, ns, no significant

Next, we verified whether CD44 mediated the impact of SRGN on microglial activation in ischemic stroke. *Cd44*-KO mice were employed, and rSRGN was injected into *Cd44*-KO mice after MCAO. The regional cerebral blood flow (CBF) was unaltered during the stroke course after *Cd44* gene knockout (Additional file 6: Fig. S4C, D). Our results demonstrated that *Cd44*-knockout could partially reversed the effects of rSRGN on infarct volume (Fig. 6A, B), neurological behavior tests (Fig. 6C, D) and production of proinflammatory cytokines (Fig. 6E, F) in MCAO mice. Moreover, the microglial morphology turned back towards a more ramified shape (Fig. 6G–I). Of note, without CD44, the significant effect of rSRGN on MCAO mice was eliminated (Fig. 6A–F). Thus, our findings showed that microglial SRGN interacted with CD44 to promote microglial activation and post-stroke neuroinflammation*.*

### SRGN activates NF-κB p65 signaling pathway in microglia

**Fig. 7:**
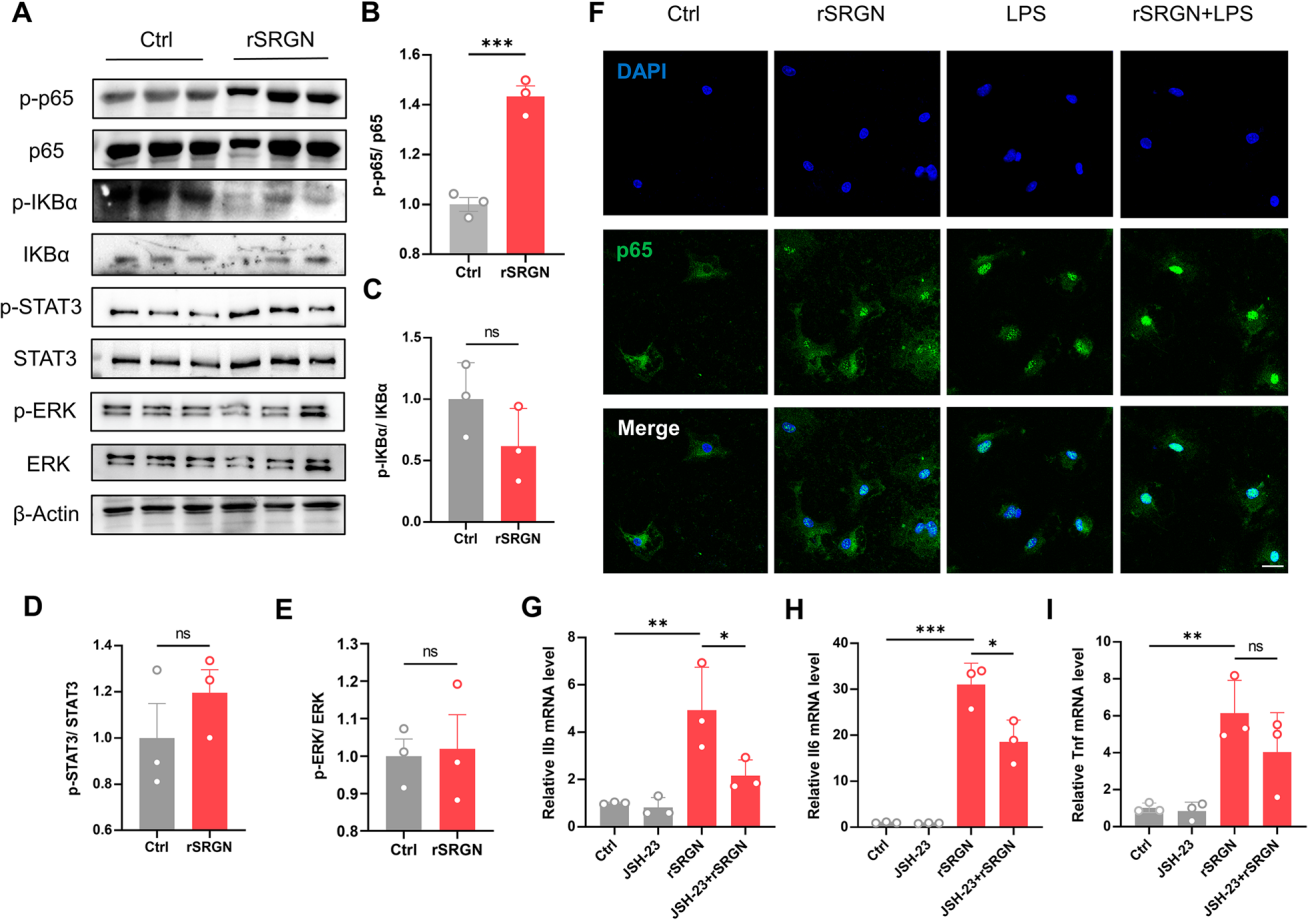
SRGN activates NF-κB p65 signaling pathway in microglia. **A-E** Representative immunoblot images (**A**) and quantification of *p*-p65/ p65 (**B**), *p*-IKB/ IKB (**C**), *p*-STAT3/ STAT3 (**D**) and *p*-ERK/ ERK (**E**) for primary microglia treated with rSRGN (50 ng/ mL) or control solvent for 6 h. β-Actin served as the loading control. *n* = 3 per group. **F** Representative immunofluorescence images showing the cellular localization of NFκB p65 (green) in primary microglia induced by rSRGN (50 ng/ mL), LPS (100 ng/ mL) or a combination of both for 3 h. Scale bar, 20 μm. **G-I** The qPCR analysis of *Il1b* (**G**), *Il6* (**H**) and *Tnf* (**I**) mRNA levels in primary microglia treated with rSRGN (50 ng/ mL, 3 h), with or without the pretreatment of JSH-23 (30uM, 2 h). *n* = 3 per group. Data represented as mean ± SEM, * *p* < 0.05, ** *p* < 0.01, ***p < 0.001, ns, no significant

Since the KEGG enrichment uncovered NF-κB signaling pathway as one of the key pathways, influenced by rSRGN, we further determined whether SRGN promote microglial activation via activating NF-κB signaling pathway. Immunoblot illustrated that rSRGN increased p-p65/p65 level, while the levels of p-IKBα/IKBα, p-STAT3/STAT3 and p-ERK/ERK were not affected (Fig. 7A–E). Meanwhile, immunofluorescence indicated that rSRGN boosted the nuclear translocation of NF-κB p65 (Fig. 7F). Moreover, we used JSH-23, a selective NF-κB p65 inhibitor [40, 41] to treat microglia before adding rSRGN. After suppressing NF-κB p65 with JSH-23, rSRGN-induced upregulation of proinflammatory cytokines were partially reversed (Fig. 7G–I). Taken together, these findings suggested the essential role of NF-κB p65 signaling in rSRGN-mediated microglial activation.

### SRGN modulates glycolysis and promotes the activation of microglia

**Fig. 8:**
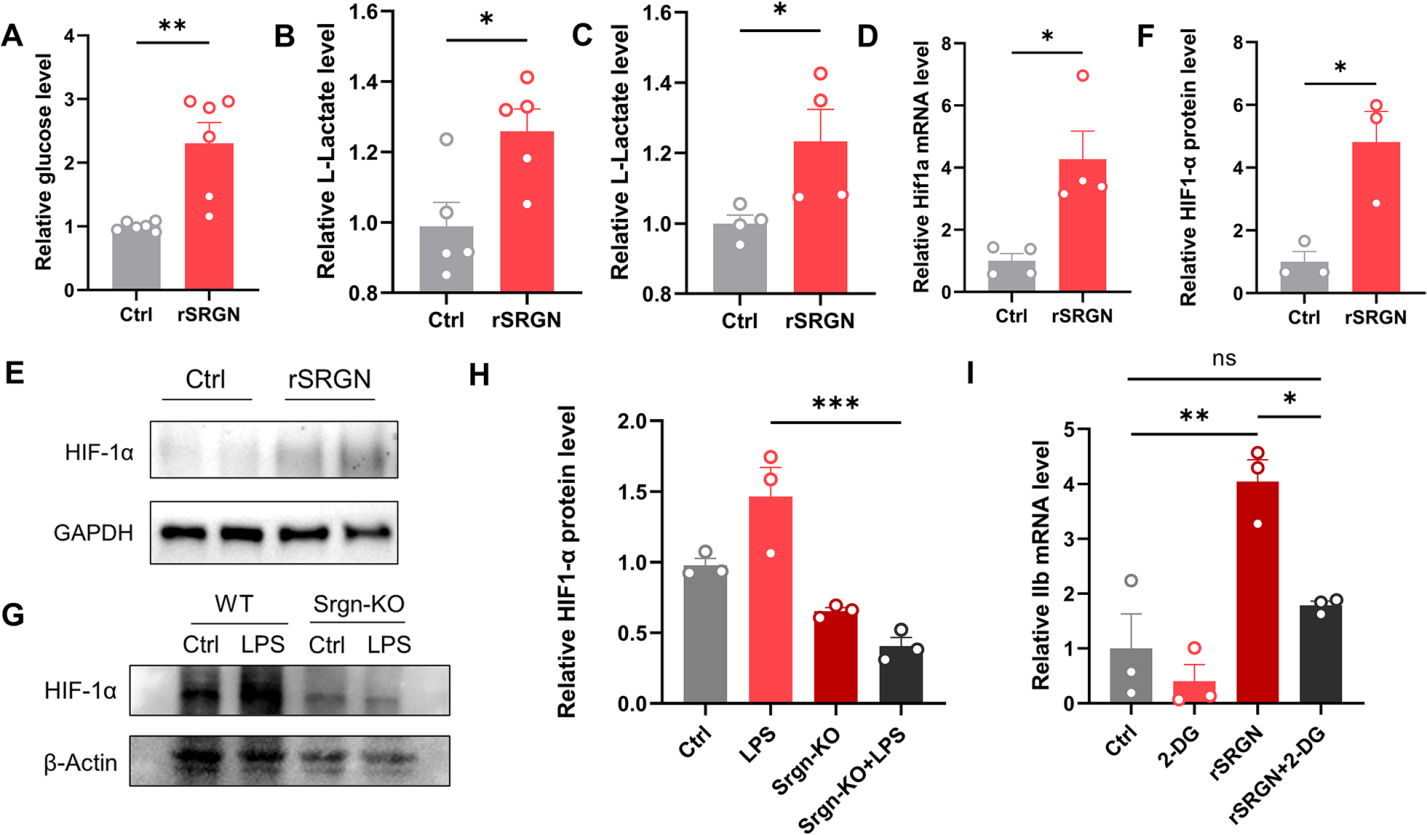
SRGN modulates glycolysis and promotes the activation of microglia. **A** Relative intercellular glucose level of BV2 cells treated with rSRGN (50 ng/ mL, 6 h) or control solvent. **B** Relative level of L-Lactate production by BV2 cells treated with rSRGN (50 ng/ mL, 6 h) or control solvent. **C** Relative level of L-Lactate in ischemic mice brain 1 day after MCAO. The mice were injected with rSRGN (2.5 mg/ mL) or control solvent soon after MCAO. *n* = 4 mice per group. **D** The qPCR analysis of *Hif1a* mRNA level in primary microglia treated with rSRGN (50 ng/ mL, 6 h) or control solvent. **E**, **F** Representative immunoblot image (**E**) and quantification of HIF-1α protein level (**F**) in microglia treated with rSRGN (50 ng/ mL, 6 h) or control solvent. GAPDH served as the loading control. **G**, **H** Representative immunoblot image (**G**) and quantification of HIF-1α protein level (**H**) in the primary microglia extracted from neonatal Srgn-KO mice and their WT littermates, with or without LPS (100 ng/ mL, 6 h) induction. β-Actin served as the loading control. **I** The qPCR analysis of *Il1b* mRNA level in primary microglia treated with rSRGN (50 ng/ mL, 6 h), 2-DG (1 mM, 2 h before rSRGN) or a combination of both. Data represented as mean ± SEM, * *p* < 0.05, ** *p* < 0.01, ***p < 0.001, ns, no significant

It was widely reported that microglial inflammation was accompanied by metabolic reprogramming from oxidative phosphorylation (OXPHOS) to glycolysis, which is vital for microglial functions [42, 43]. Thus, targeting microglial glycolysis might provide new insights into alleviating microglia-mediated neuroinflammation. CD44 was previously found to modulate energy metabolism in tumor cells [44, 45]. Therefore, we speculated that SRGN might influence the glycolytic process via CD44 and promote microglial activation. To confirm this, we firstly tested the glucose consumption level and the L-lactate (L-LA) production level in BV2 microglial cells. As a result, rSRGN significantly induced the cellular glucose content and L-LA production (Fig. 8A, B). In vivo, we also tested the L-LA level of the infarct hemisphere. As shown in Fig. 8C, mice injected with rSRGN displayed a higher L-LA production after MCAO. Next, we evaluated whether SRGN influenced glycolysis-related genes. HIF-1α is an important transcription factor in glycolysis [46, 47]. We confirmed that rSRGN induced microglial HIF1-α expression (Fig. 8D–F), while *Srgn*-knockout downregulated the expression of microglial HIF1-α under LPS stimulation (Fig. 8G, H). In addition, we used the 2-deoxy-D-glucose (2-DG), a glucose analog that can inhibit glycolysis [48] to treat primary microglia. As a result, we found that the treatment of 2-DG significantly downregulated rSRGN-induced expression of *Il1b* (Fig. 8I). In total, the outcomes demonstrated that SRGN promoted microglial glycolysis, probably through modulating HIF-1α.

## Discussion

**Fig. 9:**
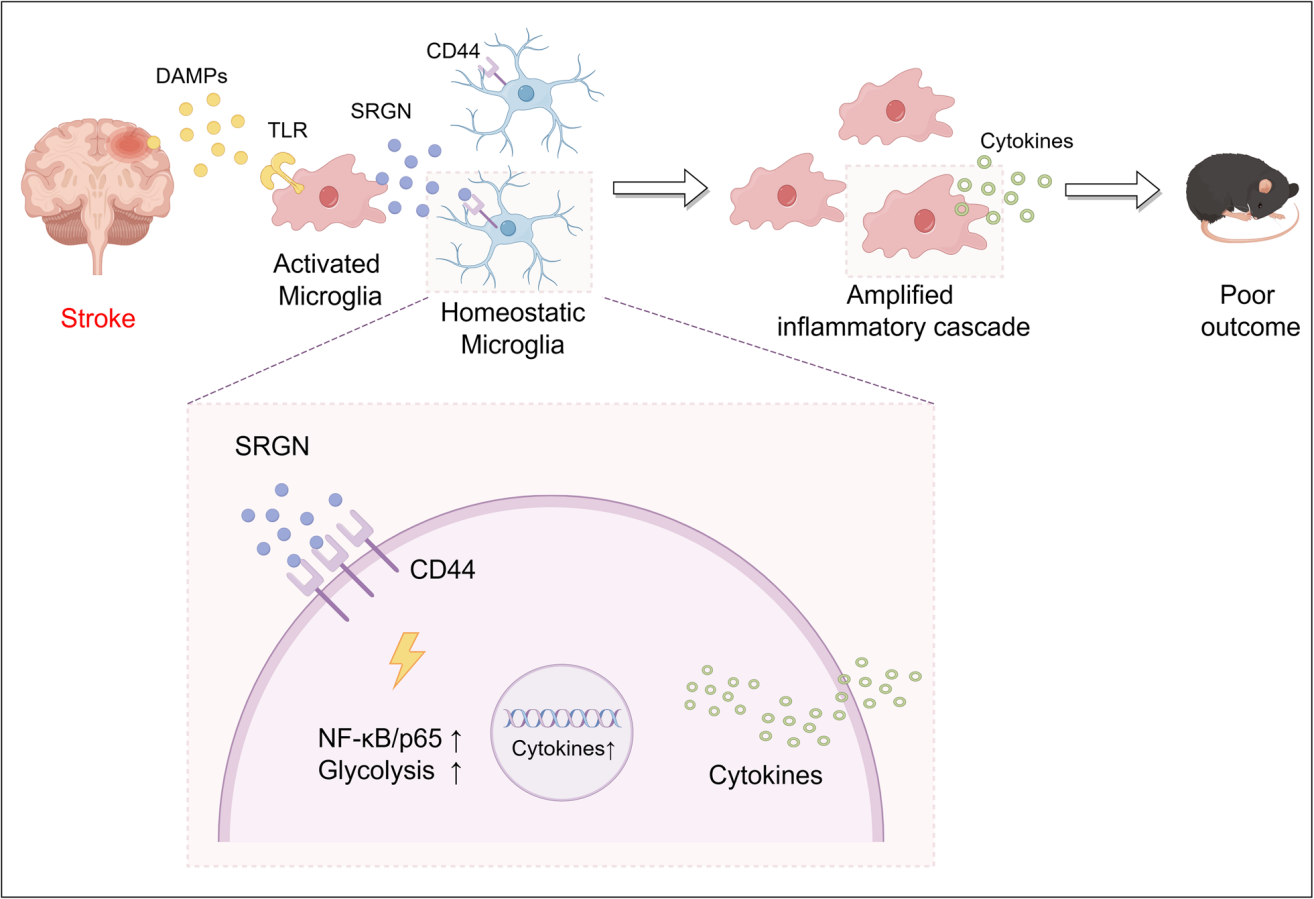
The schematic diagram of the proposed mechanisms regarding the role of microglial SRGN in ischemic stroke. By Figdraw

This study provides the first evidence that secretory protein SRGN was remarkably elevated after brain ischemia in mice. SRGN aggravated post-stroke neuroinflammation and hindered functional recoveries of ischemic stroke via promoting the microglial-mediated proinflammatory response. Mechanistically, SRGN modulated microglial activation through binding with the CD44 receptor. Moreover, SRGN activated the NF-kB p65 signaling pathway and increased glycolysis in microglia (Fig. 9).

SRGN is a dominant proteoglycan expressed in inflammatory cell types including macrophages [27], mast cells [49], chondrocytes [14], astrocytes [15] and endothelial cells [50], and the expression of SRGN is altered under different pathophysiologic conditions. However, the expression of SRGN in stroke and stroke-related microglia has not been detected. Utilizing our previous scRNA-seq data, we identified *Srgn* as one of the top DEGs in brain ischemia. In this study, we further confirmed by multiple assays (e.g., immunostaining, microglial depletion) that SRGN was significantly elevated in mouse brains after MCAO, and was mainly originated from microglia. In vitro studies illustrated that LPS but not OGD could induce the upregulation of SRGN in microglia, suggesting that microglial SRGN expression level might be sensible to DAMPs’ stimulation, but not hypoxia.

Of note, although microglia have been proved to be the main source of SRGN during the acute stages of stroke, as mentioned above, other cell types in brain (such as endothelium) and peripheral infiltrated immune cells (such as neutrophils) could still contribute to the expression and function of SRGN. Thus, the usages of global gene knockout mice may leave the interpretation of animal experiments’ results open to other cell types. In the future, the function of SRGN/ CD44 in other cell types should be studied more comprehensively and conditional knockout mice should be used if possible.

Previous studies demonstrated that SRGN take vital roles in cell proliferation, tumor migration, and inflammation [22, 51, 52]. In the neurological system, SRGN was shown to regulate the progression of schizophrenia [53], Alzheimer’s disease [54] and spine cord injury repair [15]. Among these studies, the cellular function of SRGN was investigated in astrocytes’ proliferation, maturation and neuron-related pathology. However, the regulatory function of SRGN in microglia and ischemic stroke was still undetected. Given the crucial role of microglia-mediated neuroinflammation in neurological diseases, particularly in ischemic stroke, plus our detection revealing the substantial increase in microglial SRGN expression in the ischemic brain, we further investigated the function of SRGN in post-stroke microglial activation and its influence on the outcome of ischemic stroke. We revealed that SRGN amplified post-stroke neuroinflammation via promoting microglial proinflammatory activation in MCAO mice, while *Srgn* deletion alleviated microglia-mediated neuroinflammation. Taken together, our results revealed the vital role of SRGN in exacerbating microglia-mediated neuroinflammation in ischemic stroke, suggesting the potential of SRGN to be a novel therapeutic target. However, it is worth mentioning that considering the temporal expression of SRGN after MCAO, our in vivo studies of SRGN were limited to 1 day and 3 days, leaving its function at 7 days or beyond unexplored. Future studies could focus on the long-term effects of SRGN on stroke.

Since SRGN is a secretory protein that mainly functions through binding with its receptors, how SRGN plays its function in the ischemic brain was also investigated. According to previous studies, CD44 is a well-recognized receptor of SRGN functioning in immune response [36, 55] and glucose/lipid metabolism [56, 57]. CD44 has been shown to mediate the impact of astrocytic glycoprotein on neuroinflammation in Parkinson's disease [36]. Furthermore, CD44 and its another classical ligand, hyaluronic acid (HA), are upregulated following ischemic stroke and are correlated to enhanced inflammatory response and worse functional outcomes [38, 58, 59]. Several studies have previously investigated the interaction between SRGN and CD44, mostly in cancers, such as breast cancer and non-small cell lung cancer [20, 23]. However, the interplay between SRGN and CD44 has not been explored in the context of stroke. As expected, we verified the elevation of CD44 after MCAO. As to its cellular distribution, multiple types of neural cells have been reported to express CD44, including astrocytes, oligodendrocytes and neurons [36, 58, 59]. Interestingly, in this study, we found that CD44 was mainly expressed by microglia and less expressed by other neural cells (like astrocytes) at 1 day after brain ischemia. We further confirmed the interaction of SRGN and CD44 in microglia. Our results indicated that SRGN secreted by microglia may primarily work on microglia themselves and maintain the activated state of microglia in the acute phase of ischemic stroke. Moreover, we confirmed that SRGN did exert its proinflammatory effects via CD44, as CD44 deficiency could rescue SRGN-induced poorer neurological outcomes and the exacerbated neuroinflammation in MCAO mice. Thus, by targeting SRGN and its interaction with CD44, we can gain valuable insights into treatment of stroke.

In this study, the mechanism underlying the impact of SRGN on microglial activation in ischemic stroke was further investigated. At first, our transcriptomic sequencing revealed that NF-κB signaling is a key regulator in SRGN-induced microglial proinflammatory activation. NF-κB p65 signaling is involved in the response of microglia to LPS stimulation and promotes microglia-mediated neuroinflammation. Upon activation, p65 can translocate into the cell nucleus and promote the expression of proinflammatory cytokines [60]. Here, we uncovered that SRGN triggered the activation of NF-κB p65 signaling, exhibiting a pattern similar to that of LPS. Recombinant SRGN protein induced the phosphorylation and nuclear translocation of p65. Moreover, we found that inhibiting the NF-κB p65 signaling with JSH-23 could partially blocked SRGN-induced microglial activation. Apart from this, microglial inflammation is always accompanied by energy metabolic reprogramming, shifting from oxidative phosphorylation to glycolysis. Inhibiting glycolysis could reduce microglial activation and the production of proinflammatory cytokines [42, 61]. Previous studies uncovered that CD44 participated in the modulation of energy metabolism in tumor cells. Nam et al. reported that the ablation of CD44 could induce glycolysis-to-oxidative phosphorylation transition in human breast cancer cells, with downregulation of HIF-1α and LDHA [44]. Thus, we hypothesized that SRGN might modulate microglial activation via modulating energy metabolism after binding to CD44. Consistently, our finding illustrated that rSRGN induced upregulation of glucose consumption and L-LA production in BV2 microglial cells. HIF-1α, a transcription factor induced by hypoxia, is the key element in glycolysis [62, 63]. The suppression of HIF-1α was reported to reduce microglia-associated inflammation after brain ischemia [64]. In this study, we found that rSRGN could promote HIF-1α expression and microglial glycolysis, while knockout of *Srgn* could downregulate its expression. These results confirmed our hypothesis that shift of energy metabolism state mediated SRGN-induced microglial activation, and SRGN holds potential to be a novel therapeutic target for modulating energy metabolism and activation of microglia.

As mentioned earlier, SRGN is made of a core protein and GAG chains. In this study, we mainly focused on the function of the core protein. We added exogenous recombinant SRGN to observe the influence of SRGN overload on microglial activation in vitro and in vivo, while we utilized gene knockout mice to eliminate SRGN. So far, we have proved the participation and importance of SGRN protein part in stroke pathology. But beyond this, the GAG chains of SRGN also carry valuable information and are necessary for cell migration and cancer progression [17, 22]. Lord et al. reported the binding of SRGN’s GAG chains with platelet factor 4 (PF4) in modulating cellular events in vasculature [65]. In the study of esophageal cancer [17], the researchers identified midkine (MDK) as a novel partner of SRGN’s function. Therefore, the cellular function and signaling pathways concerning SRGN’s GAG chains in ischemic stroke merits further exploration. Whether SRGN functions through receptors beyond CD44 also needs further investigation.

As a matter of fact, microglia could exhibit sex-specific phenotypes. For example, in the study of autism spectrum disorder, microglial structure, function and proteome profile were mainly altered in male mice after *Nlgn4* gene knockout [66]. In gliomas, sex-specific gene expression pattern was revealed by scRNA-seq and indicated that male glioma-activated microglia had a higher expression of MHCII encoding genes [67]. As to ischemic stroke, a sexually dimorphic disease [68, 69], sex differences in microglia-related inflammatory response were also reported [70]. However, we used only male mice in our experiments, which may limit the generalizability of our findings. Currently, the sex differences of microglial SRGN are still unclear. In the future, both male and female mice should be covered in the study of microglial SRGN after stroke to better serve the stroke treatment for both genders.

## Conclusions

In conclusion, we revealed the important role of SRGN in triggering microglial activation and amplifying neuroinflammation after ischemic stroke. We further confirmed that SRGN interacted with microglial CD44 to exert its function. Mechanically, SRGN promoted NF-κB signaling along with glucose metabolism in microglia. Thus, targeting SRGN might provide new strategies for alleviating post-stroke brain injury.

### Supplementary Information

**Additional file 1: Table S1.** List of primary antibodies used in this study.

**Additional file 2: Figure S1.** The gene knockout efficiency of *Srgn* and *Cd44*. (A) The qPCR analysis of *Srgn* mRNA level in primary microglia of *Srgn*-KO mice and their WT counterparts. (B) The qPCR analysis of *Cd44* mRNA level in primary microglia of *Cd44*-KO mice and their WT counterparts.

**Additional file 3: Table S2.** The primers used in this study.

**Additional file 4: Figure S2.** The cellular distribution and the microglial expression of SRGN. (A) tSNE plot showing the cellular distribution of Srgn gene from the ischemic brain tissue according to the scRNA-seq data from our previous work. (B) The qPCR analysis of Srgn mRNA levels in primary microglia after OGD/R.

**Additional file 5: Figure S3.** The expression of SRGN in cerebral endothelial cells. (A) Representative immunofluorescence images of SRGN expression in cerebral endothelial cells (Cd31 +) from mice after MCAO 1 day. Scale bar, 100 μm. (B) Quantification of co-localization of SRGN with Cd31 in cortex of mice 1 day after MCAO. n = 3 mice per group.

**Additional file 6: Figure S4.** The regional CBF of *Srgn*-KO mice and *Cd44*-KO mice. (A) Representative images of the regional CBF of *Srgn*-KO mice and their WT counterparts at baseline, after ischemia and reperfusion. (B) The quantification of regional CBF in (A). *n* = 4 ~ 5 mice per group. Data represented as mean ± SEM, ns, no significant. (C) Representative images of the regional CBF of *Cd44*-KO mice and their WT counterparts at baseline, after ischemia and reperfusion. (D) The quantification of regional CBF in (C). *n* = 4 mice per group. Data represented as mean ± SEM, ns, no significant.

**Additional file 7: Figure S5.** SRGN induces microglia to transit towards ischemic core-related phenotype. (A) The qPCR analysis of *Srxn1* and *Edn1* mRNA levels in primary microglia treated with rSRGN (50 ng/ mL) for 6 h. (B) The qPCR analysis of *Gpr65* and *Ms4a6c* mRNA levels in primary microglia treated with rSRGN (50 ng/mL) for 6 h. Data represented as mean ± SEM, * *p* < 0.05, *** *p* < 0.001.

**Additional file 8: Figure S6.** SRGN increased the infiltration of peripheral macrophages and neutrophils. (A) Flow cytometry strategies labeling macrophages (MoDM), neutrophils and microglia (MiDM) from ischemic brain tissue of mice 1 day after MCAO, injected with rSRGN (2.5 mg/ mL) or control solvent. *n* = 4 mice per group. (B-D) Quantification of the number of macrophages (B), neutrophils (C) and microglia (D) from the MCAO 1 day mice brains, injected with rSRGN (2.5 mg/ mL) or control solvent. Data represented as mean ± SEM, ** *p* < 0.01.

## Data Availability

The datasets used and/or analyzed during the current study are available from the corresponding author on reasonable request. The scRNA-seq data and bulk-RNA seq data from our previous work can be traced in the Sequence Read Archive (SRA) (National Centre for Biotechnology Information) by the accession number: PRJNA912889 and PRJNA809756, respectively. The RNA-seq data generated in this study can be traced by the accession number: PRJNA1045795.

## Abbreviations

SRGN: Serglycin
rSRGN: Recombinant mouse SRGN
KO: Knockout
WT: Wild-type
MCAO: Middle cerebral artery occlusion
TTC: 2,3,5-Triphenyltetrazolium chloride
mNSS: Modified neurological severity score
LPS: Lipopolysaccharide
scRNA-seq: Single-cell RNA sequencing
GAG: Glycosaminoglycan
B6: C57BL/6J
SPF: Specific-pathogen-free
OGD/R: Oxygen-glucose deprivation/reoxygenation
L-LA: L-Lactic acid
HBSS: Hanks' balanced salt solution
Co-IP: Co-immunoprecipitation
ELISA: Enzyme linked immunosorbent assay
PBS: Phosphate-buffered saline
PFA: Paraformaldehyde
OD: Optical density
DEGs: Differentially expressed genes
qPCR: Quantitative PCR
TLR: Toll-like receptor
DAMPs: Damage-associated molecular patterns
CBF: Cerebral blood flow
