# Supplementary material for: SRGN amplifies microglia-mediated neuroinflammation and exacerbates ischemic brain injury
Source: J Neuroinflammation. 2024 Jan 29;21:35. doi: 10.1186/s12974-024-03026-6 (PMC10826034; doi:10.1186/s12974-024-03026-6)
Supplement: Supplementary file 1 — Additional file 1: Table S1. List of primary antibodies used in this study. [file 12974_2024_3026_MOESM1_ESM.docx]

**Table S1.** List of primary antibodies used in this study.

| Antibodies | Host species | WB dilution | IF dilution | Companies | Catalog# |
| --- | --- | --- | --- | --- | --- |
| SRGN | Mouse | 1:1000 | 1:200 | Santa | sc-374657 |
| β-Actin | Rabbit | 1:5000 |  | Bioworld | ap0060 |
| CD44 | Rat | 1:1000 | 1:200 | Invitrogen | 14-0441-82 |
| GAPDH | Rabbit | 1:5000 |  | Bioworld | ap0063 |
| IL-1β | Rabbit | 1:1000 |  | CST | 31202s |
| TNF-α | Rabbit | 1:1000 |  | Abcam | ab183218 |
| NF-κB p65 | Rabbit | 1:1000 | 1:200 | CST | 8242s |
| p- NF-κB p65 | Rabbit | 1:1000 |  | CST | 3033s |
| IκBα | Rabbit | 1:1000 |  | CST | 9242 |
| p- IκBα | Rabbit | 1:1000 |  | CST | 2859s |
| STAT3 | Mouse | 1:1000 |  | CST | 9139s |
| p-STAT3 | Rabbit | 1:1000 |  | CST | 9145s |
| ERK | Rabbit | 1:1000 |  | CST | 4695 |
| p-ERK | Rabbit | 1:1000 |  | CST | 4370s |
| HIF-1α | Rabbit | 1:1000 |  | CST | 36169s |
| Iba1 | Goat |  | 1:500 | Abcam | ab5076 |
| GFAP | Mouse |  | 1:200 | CST | 3670s |
| GFAP | Rabbit |  | 1:200 | CST | 12389s |
| NeuN | Rabbit |  | 1:500 | Abcam | ab177487 |
