# Supplementary figures and images for: SRGN amplifies microglia-mediated neuroinflammation and exacerbates ischemic brain injury
Source: J Neuroinflammation. 2024 Jan 29;21:35. doi: 10.1186/s12974-024-03026-6 (PMC10826034; doi:10.1186/s12974-024-03026-6)

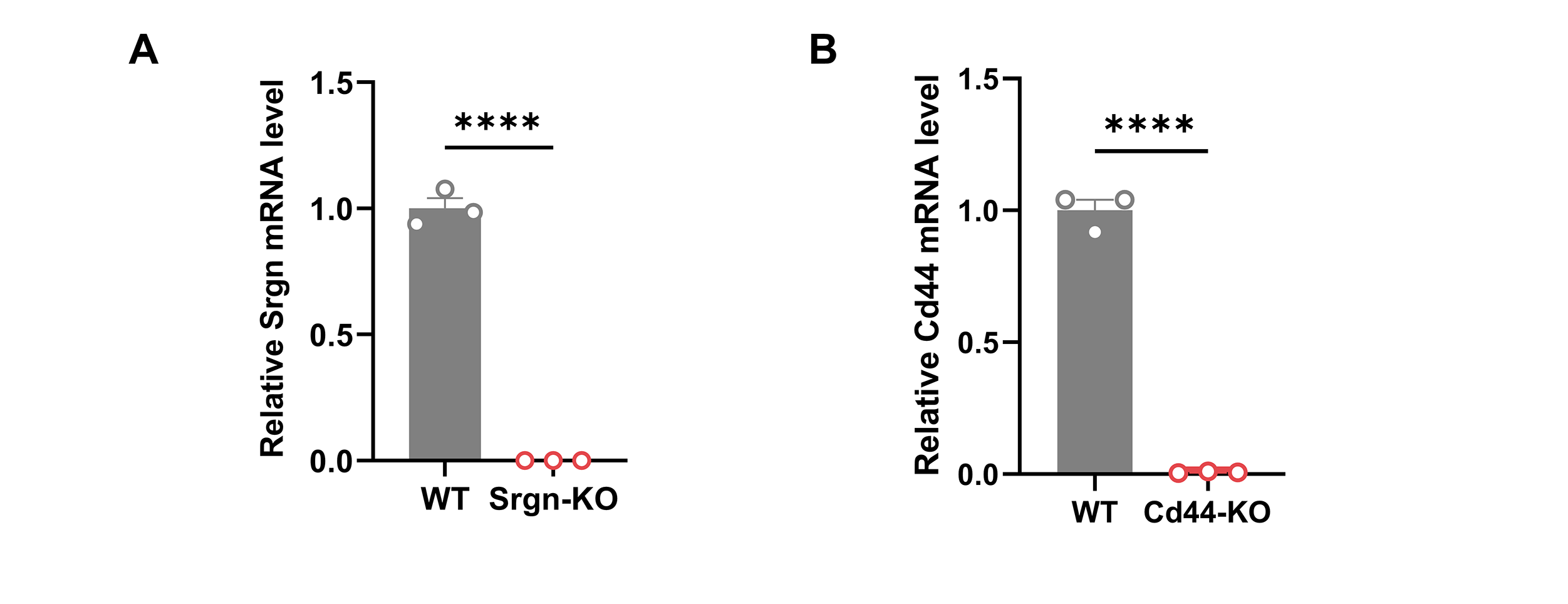

Supplement: Supplementary file 2 — Additional file 2: Figure S1. The gene knockout efficiency of Srgn and Cd44. (A) The qPCR analysis of Srgn mRNA level in primary microglia of Srgn-KO mice and their WT counterparts. (B) The qPCR analysis of Cd44 mRNA level in primary microglia of Cd44-KO mice and their WT counterparts. [file 12974_2024_3026_MOESM2_ESM.tif]

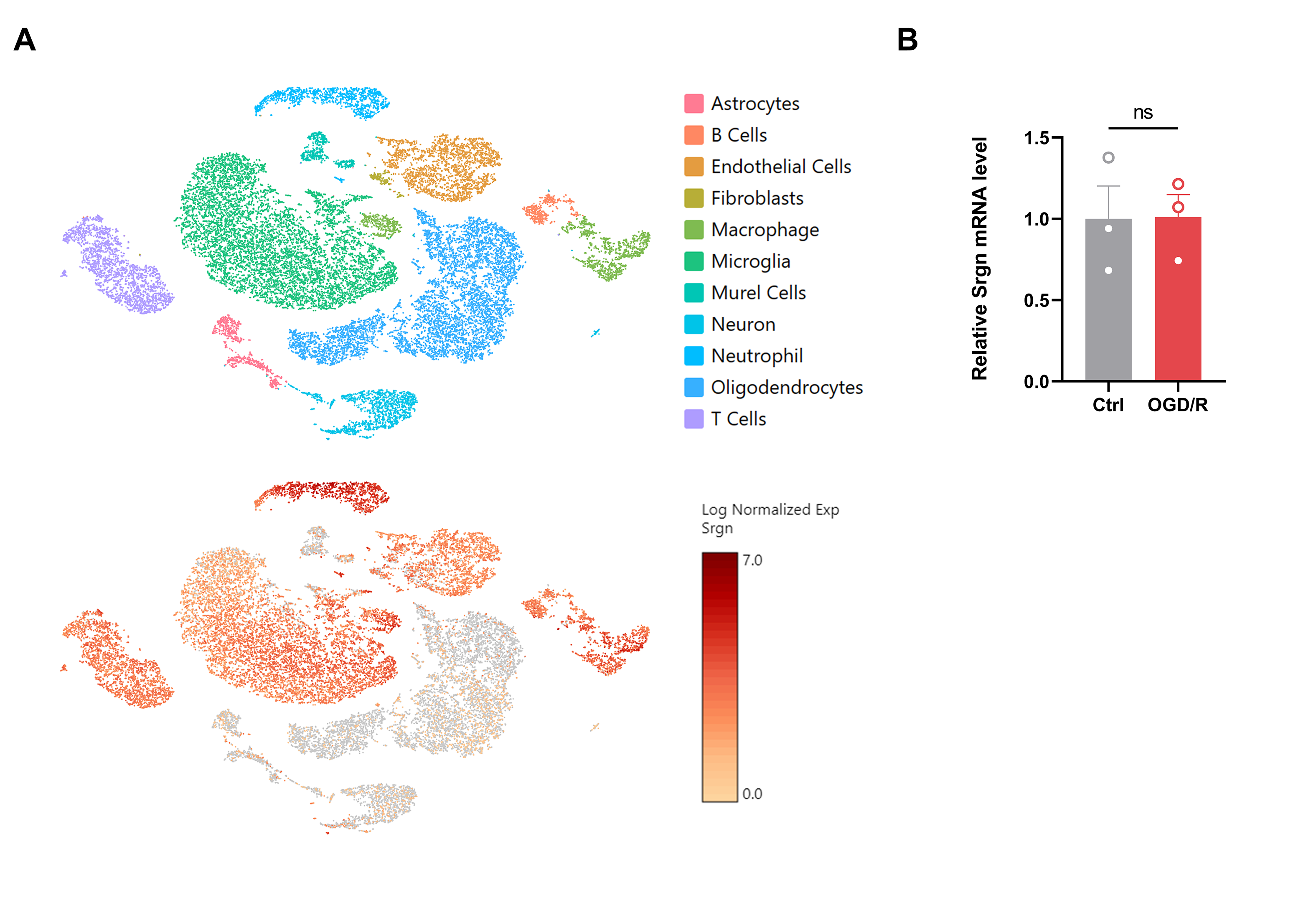

Supplement: Supplementary file 4 — Additional file 4: Figure S2. The cellular distribution and the microglial expression of SRGN. (A) tSNE plot showing the cellular distribution of Srgn gene from the ischemic brain tissue according to the scRNA-seq data from our previous work. (B) The qPCR analysis of Srgn mRNA levels in primary microglia after OGD/R. [file 12974_2024_3026_MOESM4_ESM.tif]

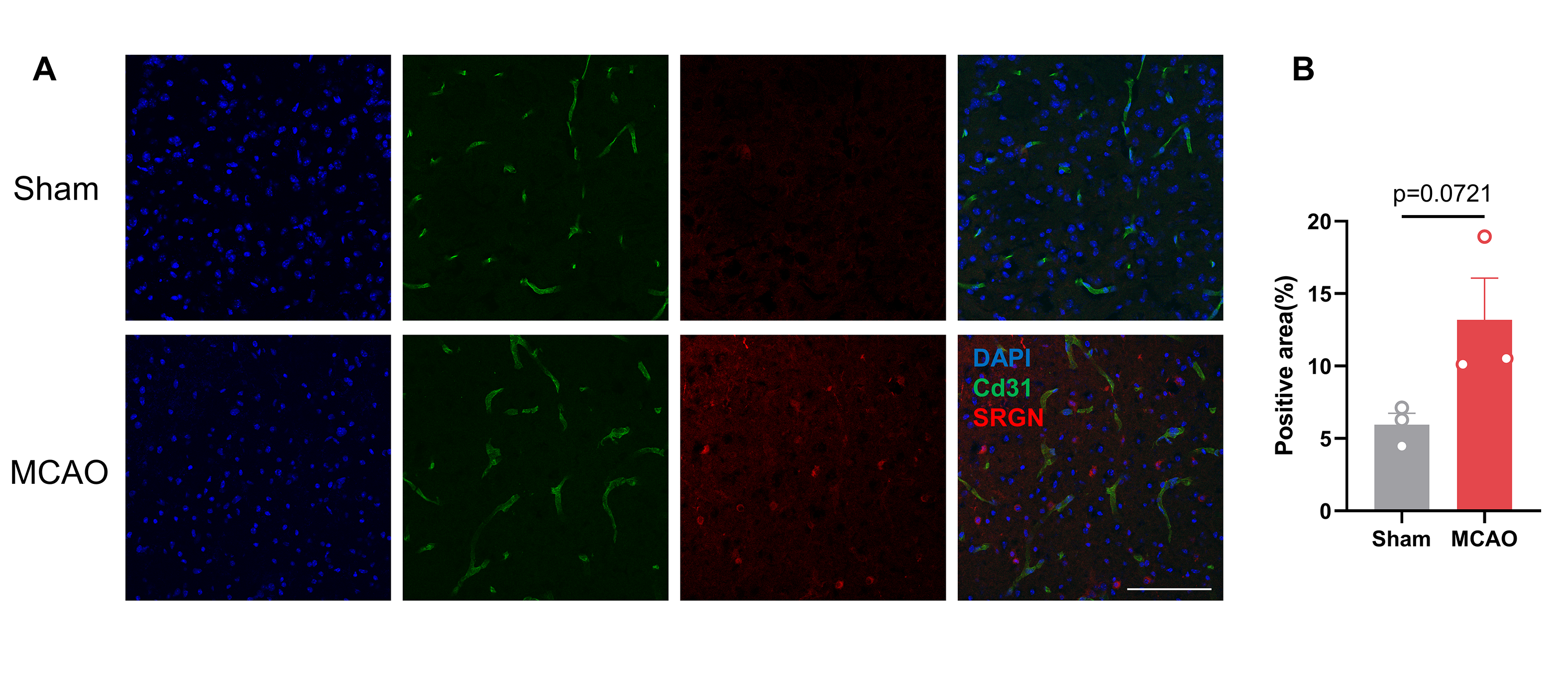

Supplement: Supplementary file 5 — Additional file 5: Figure S3. The expression of SRGN in cerebral endothelial cells. (A) Representative immunofluorescence images of SRGN expression in cerebral endothelial cells (Cd31 +) from mice after MCAO 1 day. Scale bar, 100 μm. (B) Quantification of co-localization of SRGN with Cd31 in cortex of mice 1 day after MCAO. n = 3 mice per group. [file 12974_2024_3026_MOESM5_ESM.tif]

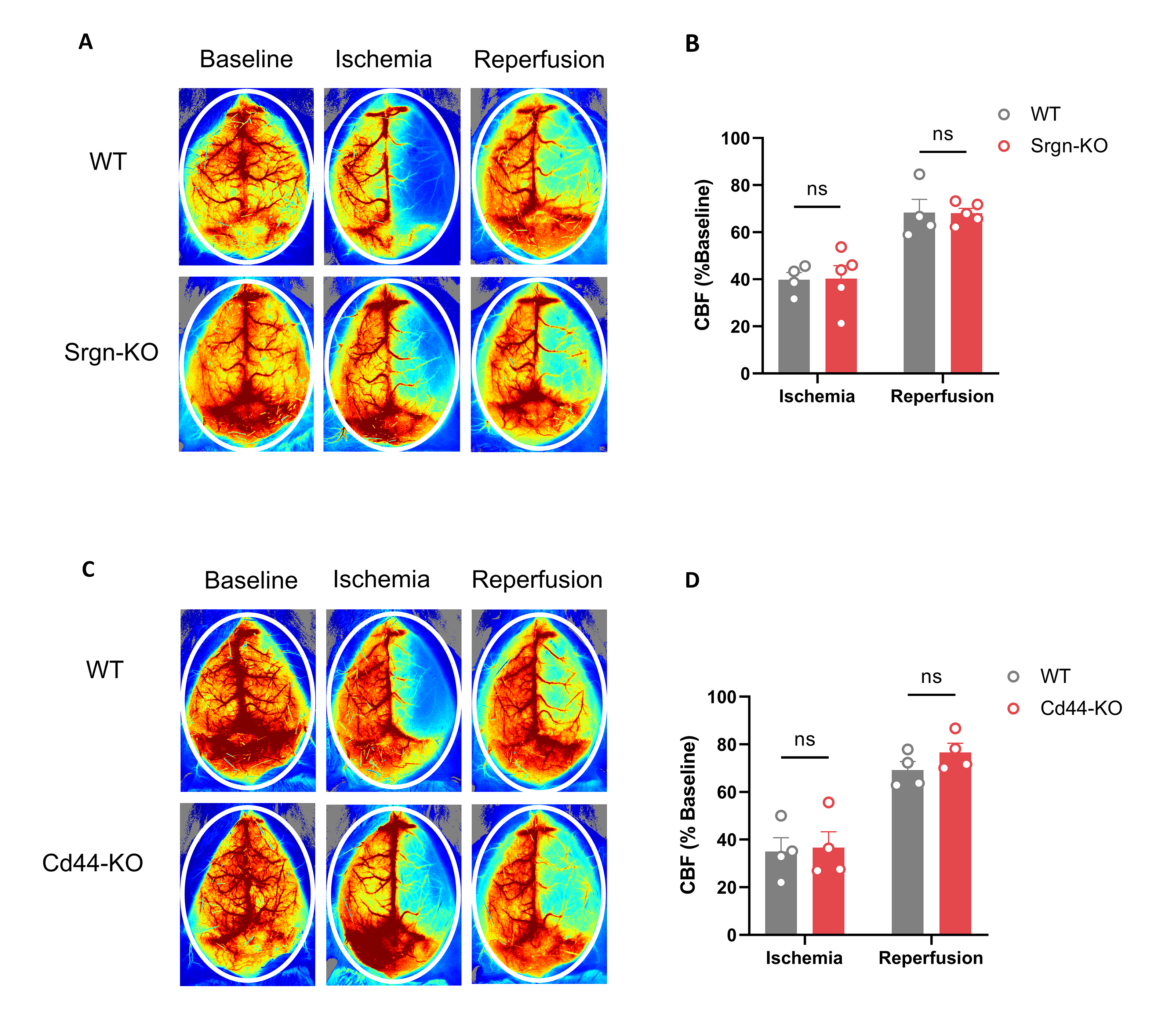

Supplement: Supplementary file 6 — Additional file 6: Figure S4. The regional CBF of Srgn-KO mice and Cd44-KO mice. (A) Representative images of the regional CBF of Srgn-KO mice and their WT counterparts at baseline, after ischemia and reperfusion. (B) The quantification of regional CBF in (A). n = 4 ~ 5 mice per group. Data represented as mean ± SEM, ns, no significant. (C) Representative images of the regional CBF of Cd44-KO mice and their WT counterparts at baseline, after ischemia and reperfusion. (D) The quantification of regional CBF in (C). n = 4 mice per group. Data represented as mean ± SEM, ns, no significant. [file 12974_2024_3026_MOESM6_ESM.tif]

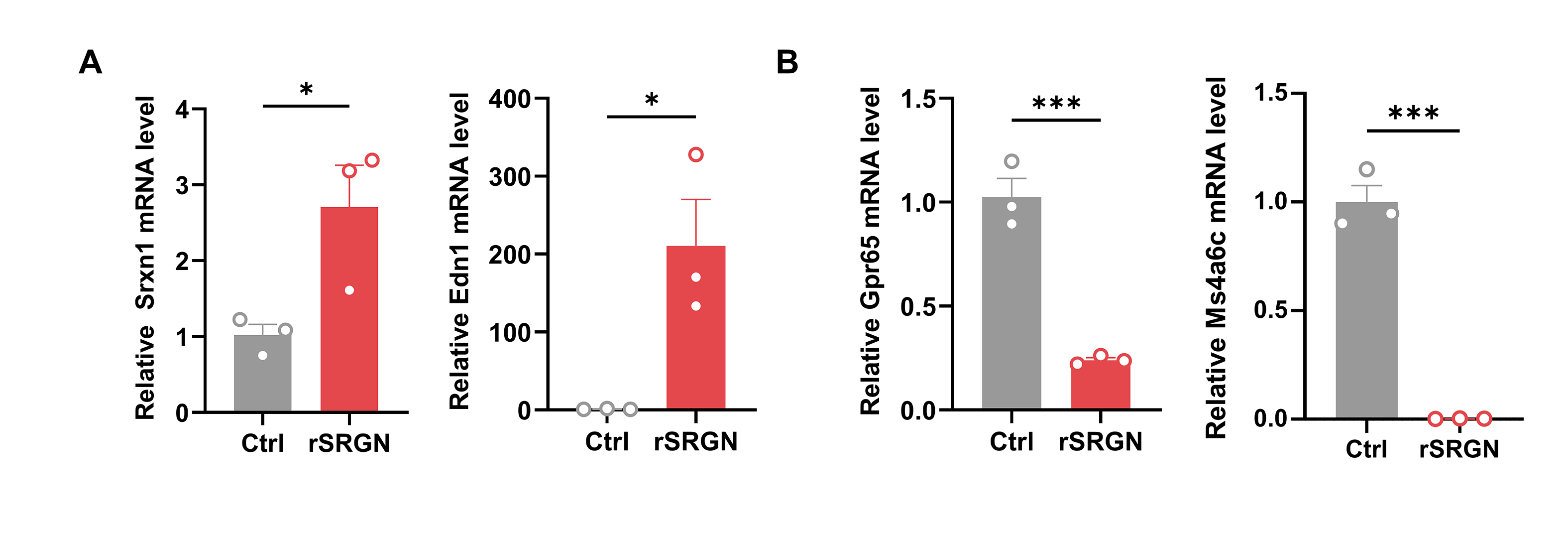

Supplement: Supplementary file 7 — Additional file 7: Figure S5. SRGN induces microglia to transit towards ischemic core-related phenotype. (A) The qPCR analysis of Srxn1 and Edn1 mRNA levels in primary microglia treated with rSRGN (50 ng/ mL) for 6 h. (B) The qPCR analysis of Gpr65 and Ms4a6c mRNA levels in primary microglia treated with rSRGN (50 ng/mL) for 6 h. Data represented as mean ± SEM, * p < 0.05, *** p < 0.001. [file 12974_2024_3026_MOESM7_ESM.tif]

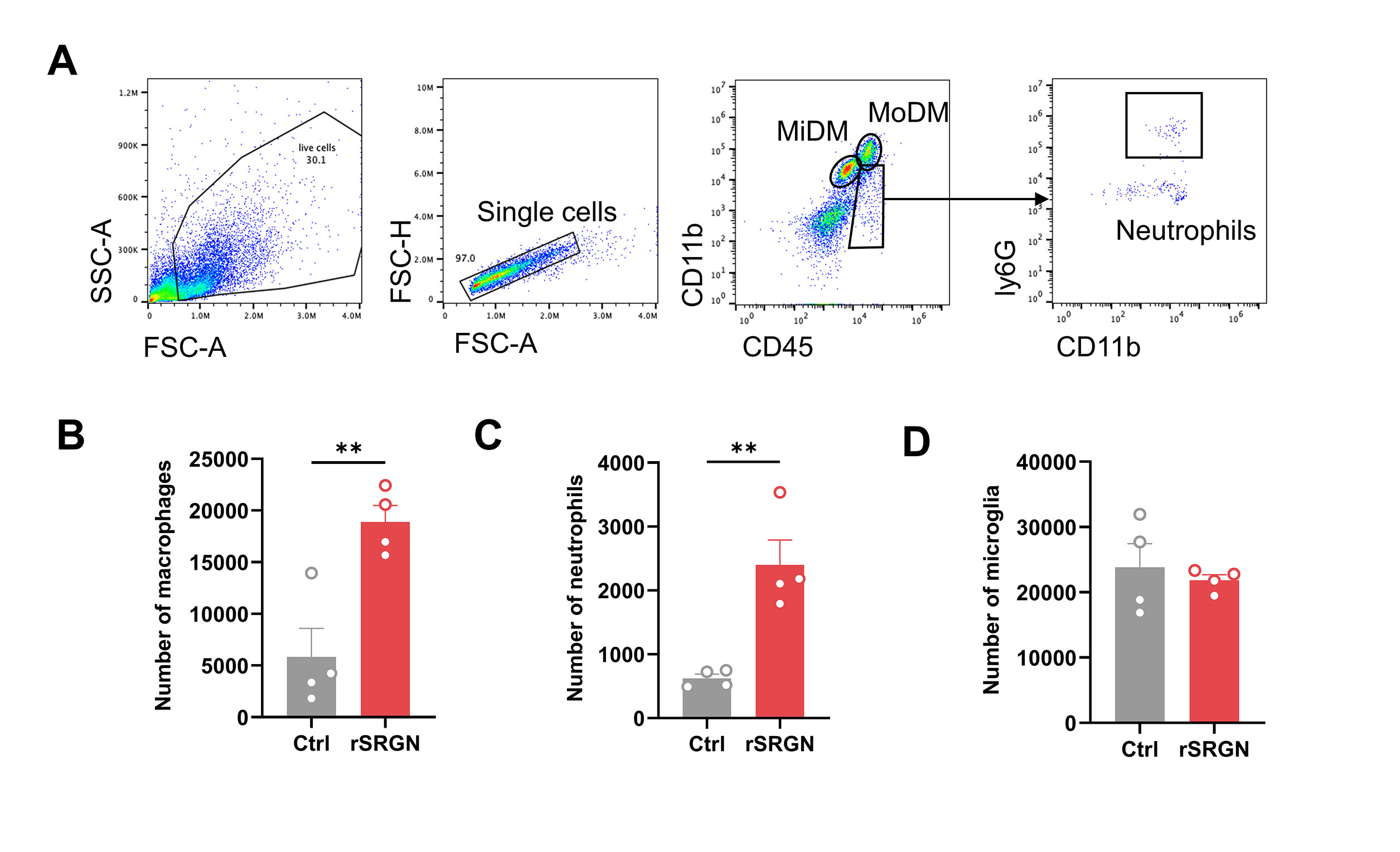

Supplement: Supplementary file 8 — Additional file 8: Figure S6. SRGN increased the infiltration of peripheral macrophages and neutrophils. (A) Flow cytometry strategies labeling macrophages (MoDM), neutrophils and microglia (MiDM) from ischemic brain tissue of mice 1 day after MCAO, injected with rSRGN (2.5 mg/ mL) or control solvent. n = 4 mice per group. (B-D) Quantification of the number of macrophages (B), neutrophils (C) and microglia (D) from the MCAO 1 day mice brains, injected with rSRGN (2.5 mg/ mL) or control solvent. Data represented as mean ± SEM, ** p < 0.01. [file 12974_2024_3026_MOESM8_ESM.tif]
