# Supplementary material for: SRGN amplifies microglia-mediated neuroinflammation and exacerbates ischemic brain injury
Source: J Neuroinflammation. 2024 Jan 29;21:35. doi: 10.1186/s12974-024-03026-6 (PMC10826034; doi:10.1186/s12974-024-03026-6)
Supplement: Supplementary file 3 — Additional file 3: Table S2. The primers used in this study. [file 12974_2024_3026_MOESM3_ESM.docx]

**Table S2.** The primers used in this study.

| Gapdh | Forward | GCCAAGGCTGTGGGCAAGGT |
| --- | --- | --- |
|  | Reverse | TCTCCAGGCGGCACGTCAGA |
| Il1b | Forward | AAGCCTCGTGCTGTCGGACC |
|  | Reverse | TGAGGCCCAAGGCCACAGGT |
| Il6 | Forward | GCTGGTGACAACCACGGCCT |
|  | Reverse | AGCCTCCGACTTGTGAAGTGGT |
| Tnf | Forward | CAAGGGACAAGGCTGCCCCG |
|  | Reverse | GCAGGGGCTCTTGACGGCAG |
| Srgn | Forward | CTCGCCTTCGTCCTGGTTT |
|  | Reverse | CCTCGATGCAGTTCGCAAAAA |
| Hif1 | Forward | ACCTTCATCGGAAACTCCAAAG |
|  | Reverse | CTGTTAGGCTGGGAAAAGTTAGG |
| Pkm2 | Forward | GCCGCCTGGACATTGACTC |
|  | Reverse | CCATGAGAGAAATTCAGCCGAG |
| Cd44 | Forward | CCACAGCCTCCTTTCAATAACC |
|  | Reverse | GGAGTCTTCGCTTGGGGTA |
| Cxcl1 | Forward | TGCACCCAAACCGAAGTCAT |
|  | Reverse | CTCCGTTACTTGGGGACACC |
| Cxcl2 | Forward | CCAACCACCAGGCTACAGG |
|  | Reverse | GCGTCACACTCAAGCTCTG |
| Cxcl10 | Forward | CCAAGTGCTGCCGTCATTTTC |
|  | Reverse | GGCTCGCAGGGATGATTTCAA |
| Nos2 | Forward | GTTCTCAGCCCAACAATACAAGA |
|  | Reverse | GTGGACGGGTCGATGTCAC |
| Ccl4 | Forward | TTCCTGCTGTTTCTCTTACACCT |
|  | Reverse | CTGTCTGCCTCTTTTGGTCAG |
| Srxn1 | Forward | CCCAGGGTGGCGACTACTA |
|  | Reverse | GTGGACCTCACGAGCTTGG |
| Edn1 | Forward | GCACCGGAGCTGAGAATGG |
|  | Reverse | GTGGCAGAAGTAGACACACTC |
| Gpr65 | Forward | ATGGCGATGAACAGCATGTG |
|  | Reverse | ACGCATAAAGATCCGATGTTGG |
| Ms4a6c | Forward | TCAAAGTGATAGTGGCAATCCAG |
|  | Reverse | CCCTTCTCTGTCTTCCCCCAT |
